# Assessing site formation and assemblage integrity through stone tool refitting at Gruta da Oliveira (Almonda karst system, Torres Novas, Portugal): A Middle Paleolithic case study

**DOI:** 10.1371/journal.pone.0192423

**Published:** 2018-02-16

**Authors:** Marianne Deschamps, João Zilhão

**Affiliations:** 1 UNIARQ – Centro de Arqueologia da Universidade de Lisboa, Faculdade de Letras de Lisboa, Universidade de Lisboa, Alameda da Universidade, Lisboa, Portugal; 2 CNRS UMR 5608 TRACES, Université de Toulouse Jean Jaurès, Toulouse, France; 3 Institució Catalana de Recerca i Estudis Avançats (ICREA), Barcelona, Spain; 4 Universitat de Barcelona, SERP (Seminari d’Estudis i Recerques Prehistòriques, SGR2014-00108), Departament d’Història i Arqueologia, Facultat de Geografia i Història, Barcelona, Spain; Max Planck Institute for the Science of Human History, GERMANY

## Abstract

We use stone tool refitting to assess palimpsest formation and stratigraphic integrity in the basal units of the Gruta da Oliveira archeo-stratigraphic sequence, layers 15–27, which TL and U-series dating places in late Marine Isotope Stage (MIS) 5 or early MIS 4. As in most karst contexts, the formation of this succession involved multiple and complex phenomena, including subsidence, bioturbation, carnivore activity and runoff as agents of potential post-depositional disturbance. During phases of stabilization, such as represented by layers 15, 21 and 22, the excavated area was inhabited and refits corroborate that post-depositional displacement is negligible. Layers 23–25 and 16–19 correspond to subdivisions that slice thick geological units primarily formed of material derived from the cave’s entrance via slope dynamics. Refit links are consistent with rapid fill-up of the interstitial spaces found in the Karren-like bedrock (for layers 23–25), or left between large boulders after major roof-collapse events (for layers 16–19). Layers 26 (the “Mousterian Cone”) and 27 are a “bottom-of-hourglass” deposit underlying the main sedimentary body; the refits show that this deposit consists of material derived from layers 15–25 that gravitated through fissures open in the sedimentary column above. Layer 20, at the interface between two major stratigraphic ensembles, requires additional analysis. Throughout, we found significant vertical dispersion along the contact between sedimentary fill and cave wall. Given these findings, a preliminary analysis of technological change across the studied sequence organized the lithic assemblages into five ensembles: layer 15; layers 16–19; layer 20; layers 21–22; layers 23–25. The lower ensembles show higher percentages of flint and of the Levallois method. Uniquely at the site, the two upper ensembles feature bifaces and cleavers.

## Introduction

Even though stone tool refitting goes back to the end of the 19th century [1–2], it is only since the 1960’s that it has been widely used to reconstruct past technological systems and assess assemblage integrity and site formation process. Building on the “*chaîne opératoire*” concept derived by Leroi-Gourhan [3] from Mauss’s [4–5] ethnographic “*chaîne technique*” [6], stone tool refitting was systematically applied for the first time at Pincevent (Seine et Marne, France), a site whose pristine conservation of occupation surfaces represented an ideal scenario for the application of the technique [7]. Core reduction sequences and the economy of debitage products were reconstructed in detail, and the distribution of refitted elements was used to make inferences about the contemporaneity of different loci, the spatial organization of activities, or the social structure of the hunter-gatherer bands that had discarded the analyzed remains [8–10]. The Pincevent example has since been emulated at sites presenting a similar degree of preservation [11–12], while the analysis of vertical distributions has been used to assess the impact of palimpsest formation and post-depositional disturbance at both open-air and cave or rock-shelter stratified sites [13–22].

With regards to the Middle Paleolithic, most refitting studies have so far concerned sites in the open-air [23–31], even though a few examples of its application in rock-shelter contexts also exist [16,32–39]. Here, we add to this limited corpus a case study from Gruta da Oliveira (Almonda karst system, Torres Novas, Portugal). This site contains a ca.13 m-thick stratigraphic succession rich in Middle Paleolithic stone tools and bone remains, including human ones, and is dated by a combination of different methods to the interval between ca.35 and ca.105 ka (thousands of years) [40–43]. Our study targeted the basal part of the sequence, layers 15–27. As implied by the minimum age imposed by the TL dating of overlying layer 14 to 78±8 ka [43], these basal units formed during early MIS (Marine Isotope Stage) 4 or late MIS 5.

Based on the observations made during excavation, coupled with the micromorphological analysis of stratigraphic thin sections, the geoarcheological study of the deposit concluded that the accumulation of layers 15–27 had proceeded through roof and wall collapse episodes alternating with phases of stabilization [40]. The presence throughout of subsidence-deformation features and of roots and animal burrows, indicated, however, that a degree of post-depositional disturbance was to be expected, while long-distance vertical displacement along voids between the cave walls and the sedimentary column represented an additional cause of potential contamination (by material derived from higher up in the sequence).

A refitting study carried out on the lithic assemblages from the upper part of the sequence (layers 8–14) had shown the impact of these processes to be limited [44], but the issue remained to be assessed for the lower part. A systematic intra- and inter-level refitting project was therefore designed as a prerequisite to the techno-economic analysis of the collections from layers 15–27. The aim was to define and constitute the analytical units best suited for the study of the stone tool assemblages in all possible dimensions, e.g., raw-material provenience and selection, technology, typology, debitage economy, use-wear, and spatial distribution.

The questions of wider methodological interest that we addressed are as follows:

- Do the archeo-stratigraphic units defined at the time of excavation constitute valid units for the organization of the stone tool collections into behaviorally meaningful assemblages, or do those units need to be grouped, or further subdivided, and, if so, how?
- Which parts of the excavation trench were most affected by post-depositional disturbance, how significant was such an impact, and, if significant, how do we separate the reliable from the unreliable units of provenience, and how is such a separation affected by the variation observed along the sequence in the topography of the encasing bedrock?
- Which of the meaningful/reliable assemblages produced by the application of the taphonomic filters are amenable to paleo-ethnographic analysis of activity (those with limited palimpsest formation and attendant good preservation of the distributions’ original spatial structure), which can only lend themselves to analysis of long-term processes (those with significant palimpsest formation and attendant loss of the distributions’ original spatial structure), and which conclusions of chrono-stratigraphic significance can be drawn from the changes observed through time in the composition of the assemblages?

For most of its duration, the Middle Paleolithic of Europe falls outside the limits of applicability of radiocarbon, and alternative dating methods are of much lower precision. Thus, the period’s chronological structure remains largely based on the patterns of change in technology and typology observed across reference sequences, and in the correlation of such sequences using lithic assemblage composition patterns coupled with paleoenvironmental information [45–48]. The potential impact of functional variability on such patterns has long been the object of much debate (e.g. [49–50]), but the extent to which the units of analysis used in the different schemes that have been proposed are indeed stratigraphically valid has often been overlooked. Yet, in stratified sites of the cave and rock-shelter type, post-depositional disturbance and palimpsest formation are bound to have impacted assemblage composition—and the more so in periglacial contexts. We believe, therefore, that the approach we used to tackle these problems at Gruta da Oliveira is of wider interest and, hence, justifies dedicated publication of our methodology and results.

## Materials and methods

### The site

**Fig 1:**
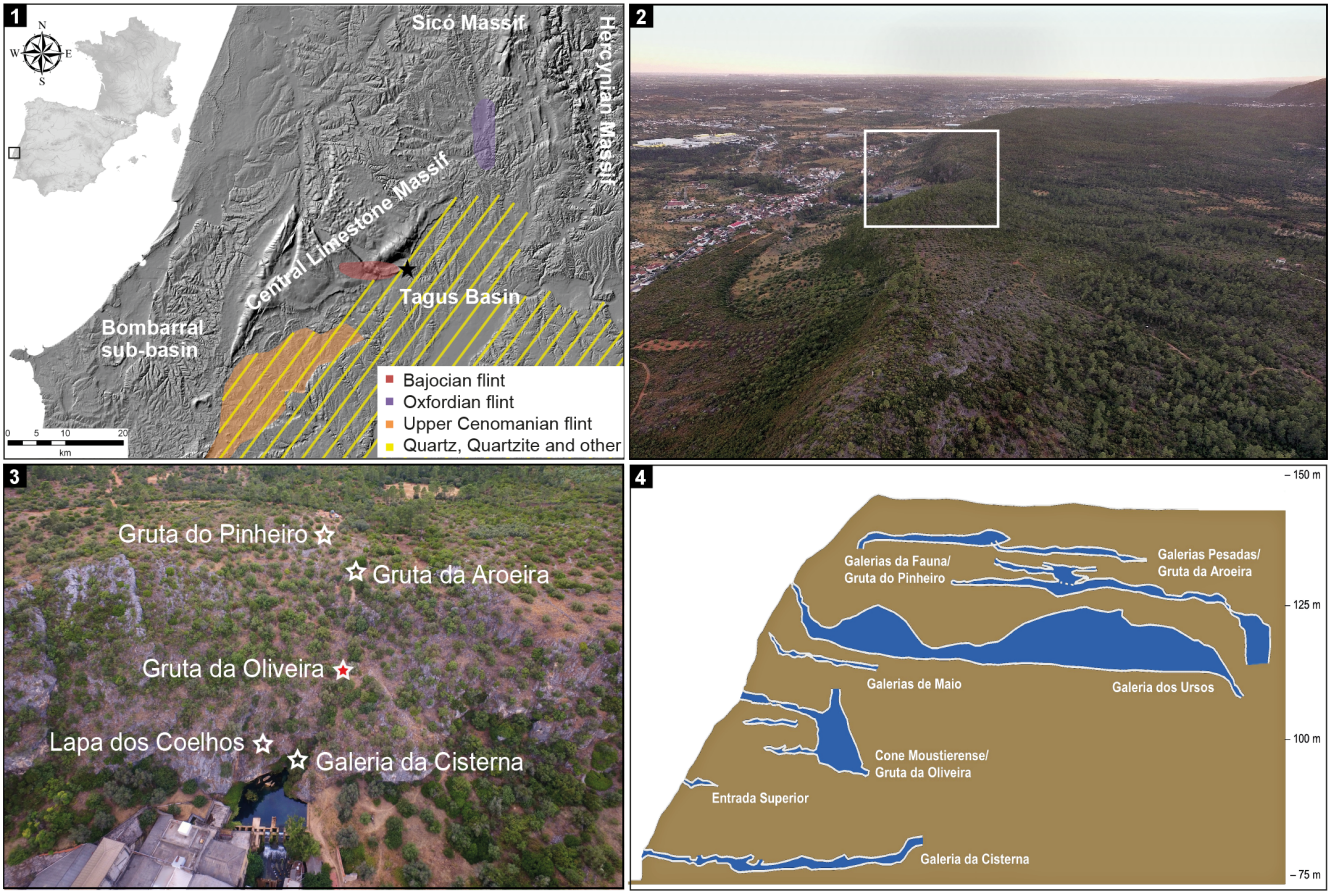
The Almonda karst system. **1**. Geographical location and raw-material sources (reprinted from [59] under a CC BY license, with permission from Journal of Lithic Studies, original copyright 2016). **2**. Drone view from NE over the fault escarpment separating Portuguese Estremadura’s Central Limestone Massif from the Cenozoic basin of the Tagus; the rectangle indicates the position of the network of underground passages associated with the spring of River Almonda. **3**. Drone view from the South over the cliff face above the spring showing the position of the currently known cave entrances that have been archeologically excavated (Galeria da Cisterna and Lapa dos Coelhos—Later Prehistory, Neolithic, and Upper Paleolithic; Gruta da Oliveira—Middle Paleolithic; Gruta da Aroeira—Acheulean; Gruta do Pinheiro—Middle or Lower Paleolithic hyena den). **4**. Schematic cross-section of the Almonda cliff face along the main fault, with indication of the archeological localities currently known in the staircase of fossil passages above the spring.

The spring of River Almonda is a karst outflow located at the base of a ca.70 m-high cliff face, part of the NE-SW, ca.40 km-long fault escarpment that separates Portuguese Estremadura’s Central Limestone Massif form the Cenozoic basin of the Tagus. Due to the tectonically-induced rising of the massif relative to the basin, the underground sections of the river underwent a process of downward migration in the karst network that left behind, at higher elevation, an interconnected, labyrinthine system of passages leading to fossil springs that once functioned as cave entrances. With time, these entrances filled-up with sediment, collapsed, and became covered by slope deposits. A program of systematic speleo-archeological survey of the system initiated in the late 1980s has since discovered, re-exposed and excavated a few [51–52] (Fig 1). Gruta da Oliveira, whose entrance lies at an elevation of ca.115 m, was the first such collapsed cave to be found—in 1989—and investigated—over some 20 field seasons ending in 2012 [40–43,53–59].

**Fig 2:**
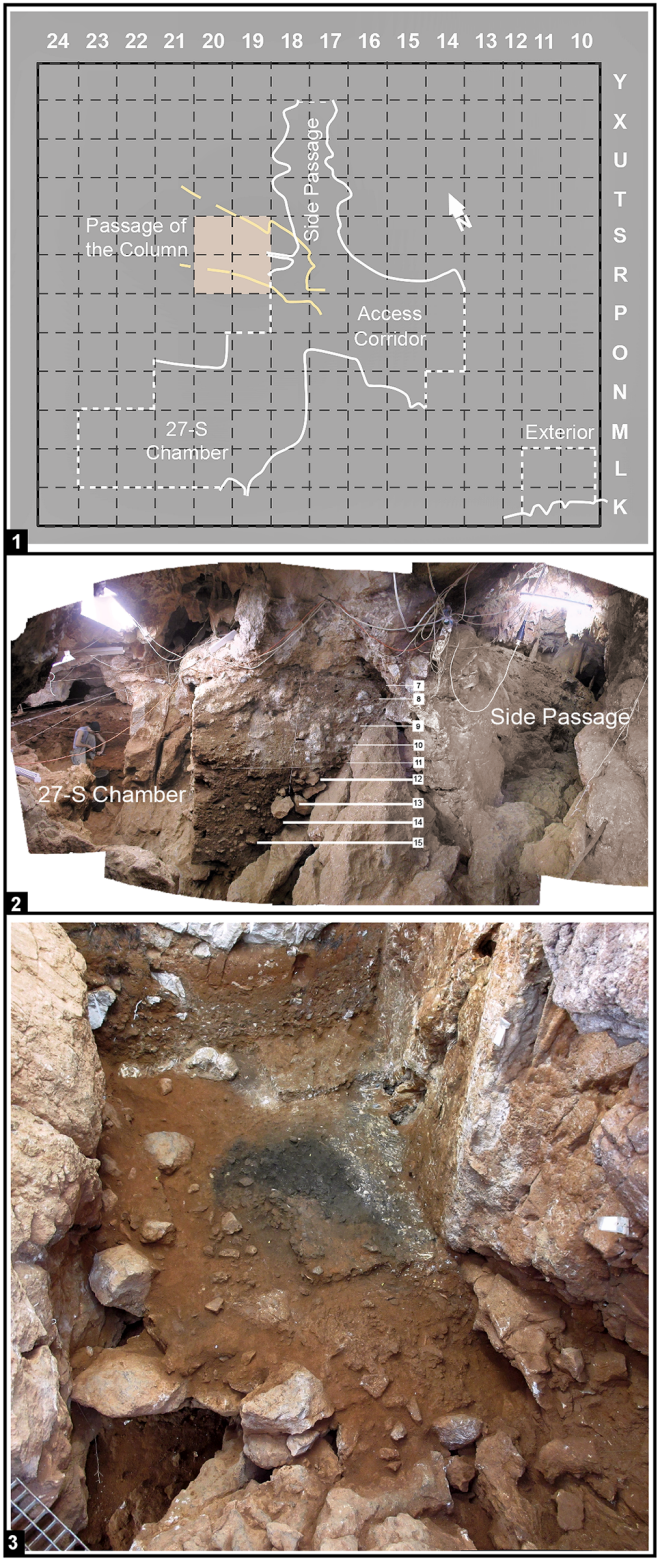
Gruta da Oliveira: Site layout and excavation views. **1**. Plan and excavation grid; wall contours are shown at the elevation of layer 10; the colored grid units indicate the position of the initial findspot, the “Mousterian Cone,” a deposit underneath the sedimentary column that, at the time of discovery, blocked the Passage of the Column. **2**. View from the Access Corridor onto the two branches at the back end of the cave, the 27-S Chamber and the Side Passage; the archeo-stratigraphic units (layers) differentiated in these sections of the site are indicated. **3**. View over the Access Corridor from its back end taken during the excavation of spit A67 (layer 21); note the voids along the contact between sedimentary column and cave walls, the hearth feature in the middle of the trench, and, bottom left, the direct connection with the Mousterian Cone and the Passage of the Column beyond.

Going in from the re-opened entrance, the Gruta da Oliveira begins as a now unroofed porch area, the “Exterior,” leading to a short, meandering passage developed along the fault through which the Almonda progressively incised its underground course. This “Access Corridor” is, on average, ca.10 m-high and 3 m-wide. Some 10 m inward from the collapsed entrance, the Access Corridor branches into three sections that, even though located at different elevations, remain linked by narrow fissures and joints: at the bottom, the “Passage of the Column” (*Galeria da Coluna*), which communicates the cave with the “Passage of the Sieve” (*Galeria do Crivo*) and the depths of the endokarst beyond; higher-up, to the left, the “September 27 (27-S) Chamber” (*Sala 27 de Setembro*), a low and wide space (ca.4×4 m and, on average, ca.2.5 m-high) developed along a stratification joint perpendicular to the main fault; and, at the top, to the right, the “Side Passage” (*Divertículo*), a narrow conduct that leads to the Passage of the Sieve through a different underground path (Fig 2).

The minimal unit used in the excavation of the Gruta da Oliveira was the “spit” or *décapage*. Spits are slices of the deposit differentiated to constrain the horizontal and vertical position of non-piece-plotted or sieve-collected finds. Spit thickness varied between 15 to 20 cm, when going through levels primarily made of large éboulis, and 5 cm, when dealing with finer sediments with potentially good preservation of spatial distributions. In all cases, the delimitation of a spit’s basal surface followed the general dip of the stratification and respected observed stratigraphic boundaries whenever they imposed a reduction of the originally targeted thickness. From top to bottom of the deposit, spits were numbered sequentially, per square meter unit of the grid or per unit of open-area excavation, from 1 to *n*, preceded by the letter A (e.g., spit A1, spit A70, etc.).

At the time of excavation, spits were grouped into “layers,” defined on the basis of features that could be followed across significant horizontal extents and were suggestive of change in the dynamics of the accumulation, such as: base-of-boulder planes defining the cave floor extant at the time of roof-collapse events; flowstone or calcite-incrusted surfaces; increases in the clay content of the matrix; and associated color changes. As some of these “layer” units span the three main sections of the cave plan (Access Corridor, 27-S Chamber, and Side Passage), the different sequences excavated in each section could be laterally correlated and integrated into a single stratigraphic succession scheme [40].

**Fig 3:**
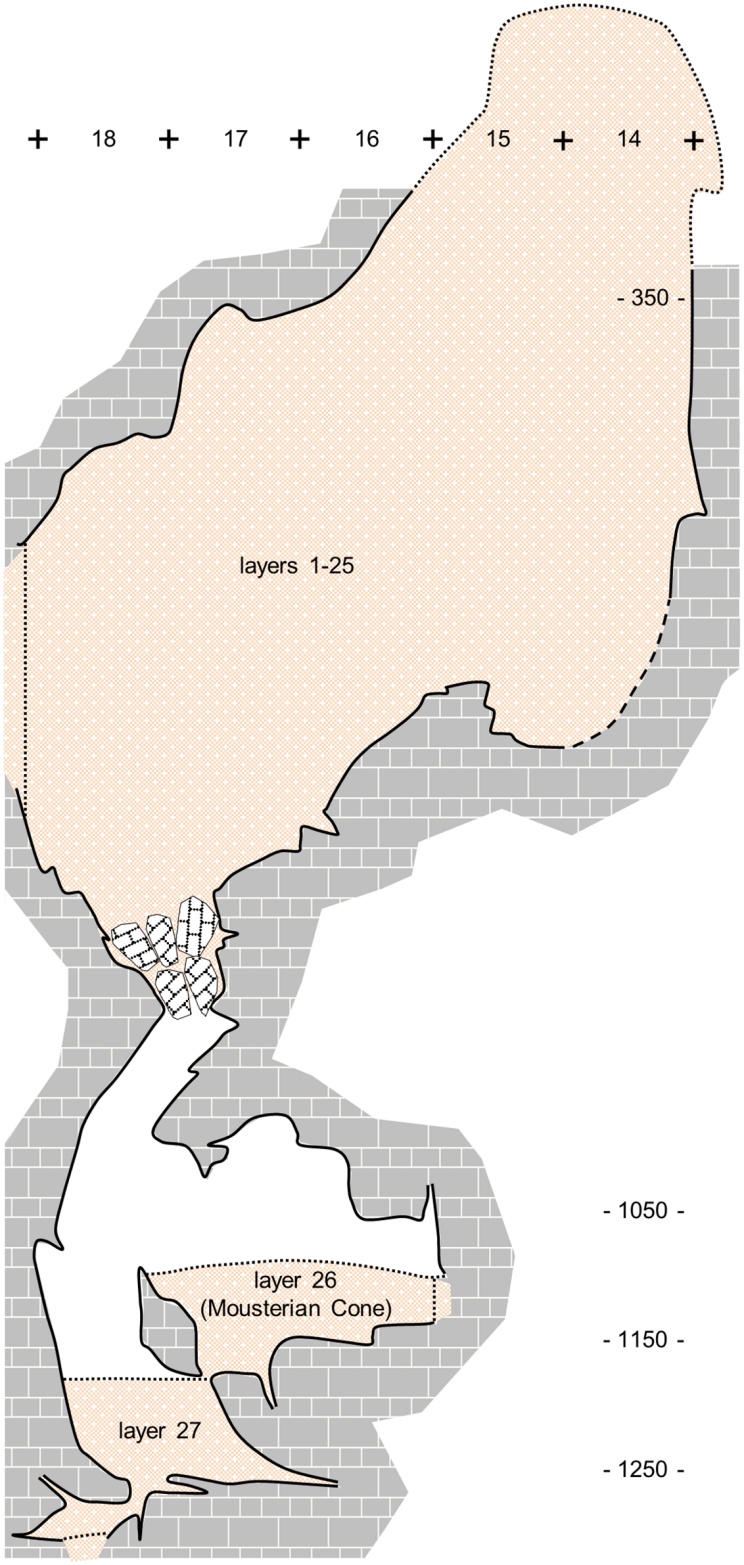
Gruta da Oliveira: Topographic cross-section along the intersection between columns P and R of the grid. Note the complete colmatage of the cave by the sedimentary column. The Mousterian Cone (layer 26) is a “bottom-of-hourglass” deposit filtered down through voids in the basal éboulis and along the cave walls that also fills the Karren-like bedrock of the Passage of the Column below (layer 27). Elevations are in cm below site datum.

**Fig 4:**
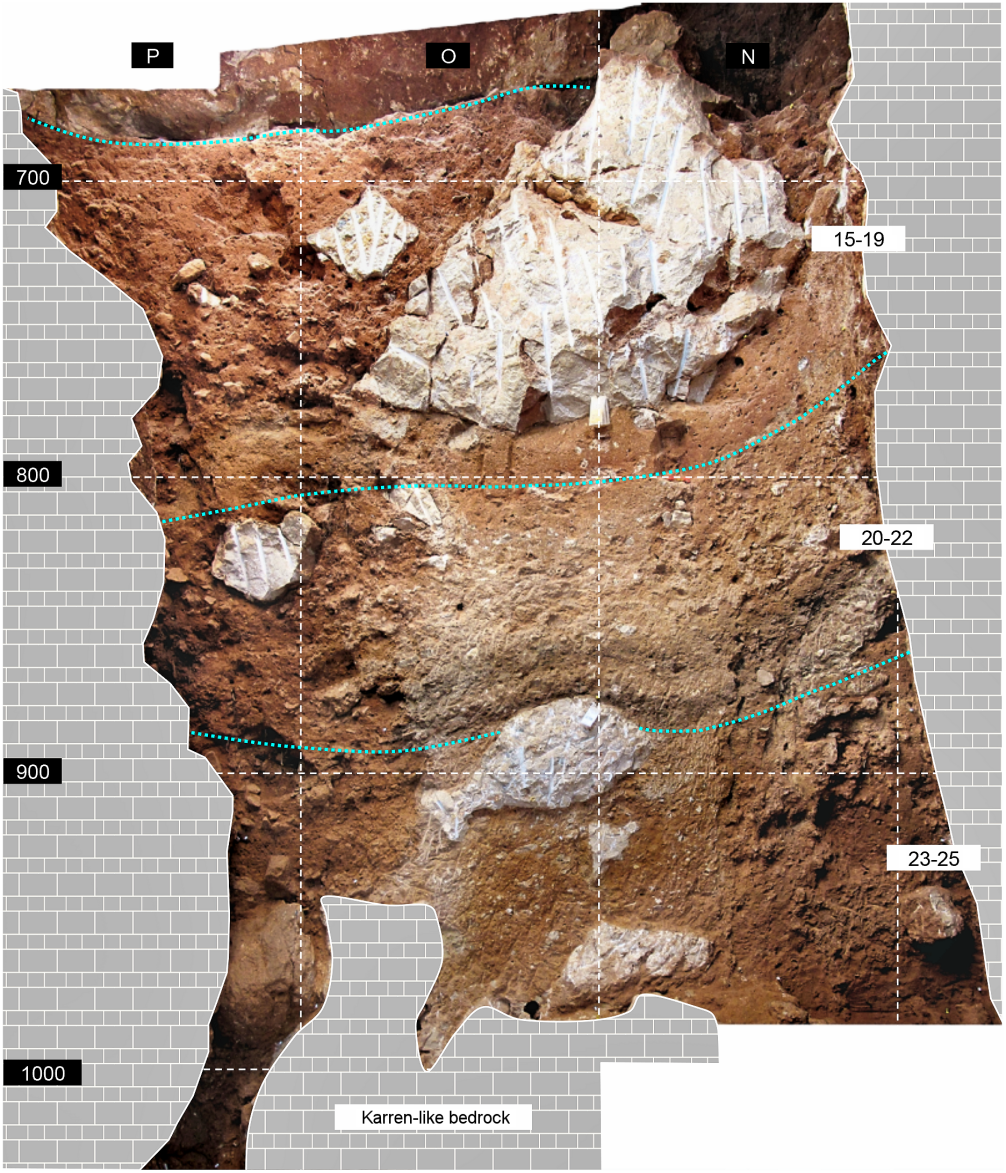
Gruta da Oliveira: The reference cross-section (N-P15>14) for the Access Corridor’s lower levels. The dotted lines indicate the boundaries between the three geo-stratigraphic ensembles recognized here. The subsidence-deformed dark bands at the base of layer 22 represent the edges of large hearth features extending outward beyond the trench wall. Elevations are in cm below site datum.

The Gruta da Oliveira layers are the equivalent of the Geoarcheological Field Unit (GFU) as theoretically defined in the context of the excavation of the Lagar Velho rock-shelter (Leiria, Portugal) [60]: a three-dimensional body composed of sediments, of either natural or cultural origin, that differ from the surrounding ones in patterned manner. Once bedrock was reached and the excavation completed, the comprehensive view of the succession thusly obtained led, however, to the grouping of the excavation’s layers/GFUs into ensembles that come closer to true stratigraphic units in the geological sense. As currently recognized, based on Zilhão et al. (2013), such ensembles can be described, from bottom to top, as follows (Figs 2–4).

#### Passage of the Column and Mousterian Cone (layers 26–27)

Basal units of the succession, the initial discovery of which, in 1989, triggered the site’s subsequent excavation. The Mousterian Cone was an éboulis cone with a silty-sand matrix that blocked the speleological progression towards the outside for cavers approaching the cliff face from the Passage of the Sieve. Fossilized remains of a Pleistocene fauna associated with Middle Paleolithic stone tools with no signs of transport-damage were observed on the surface, and its quarter cubic-meter test excavation, carried out in 1990, yielded some 200 stone tools and 150 bones and bone fragments, 30% of which burnt [52]. This cone was interpreted as a “bottom-of-hourglass” deposit whose matrix and archeological components had filtered down through fissures linking it to an archeological cave fill inferred to exist above. The inference led to the search for the cave’s entrance, eventually found, re-opened and excavated—the Gruta da Oliveira. When reached from the top, in 2012, the Mousterian Cone was found to be the equivalent of layer 26 of the overall succession, from which, however, it was largely disconnected. At that time, sediment was also observed in fissures at the base of the Mousterian Cone area; forming the lateral equivalent of the Passage of the Column’s fill, that deposit was excavated as layer 27.

#### Access Corridor Lower Ensemble (layers 23–25)

Large rock masses stuck sub-vertically between wall and wall, supporting the overlying stratification, and roofing the empty space at the end of the Passage of the Column that enabled the original detection of the Mousterian Cone (Fig 2, panel 3). A degraded stalagmitic crust separated the upper 25 cm of this ensemble (layer 23) from the ca.65 cm (layer 24) that remained until bedrock was reached in column N of the grid. Layer 25 was mostly the éboulis, ca.80-90 cm-thick, that, in columns O-P of the grid, filled, down to the elevation of the Mousterian Cone’s surface, the narrow space communicating the Access Corridor with the Passage of the Column. The fine matrix between boulders and blocks contained a low-density scatter of bones and artefacts distributed across the ca.1.5 m thickness of the deposit but mostly coming from column P, which was significantly affected by roots and featured significant voids along the contact between sediment and cave wall (Fig 4). Burnt flints and burnt bone were found in layer 23 but, given the evidence for post-depositional disturbance, were considered at the time of excavation as probably related to the hearth features found in layer 22 above.

#### Access Corridor Middle Ensemble (layers 20–22)

Silty loam, ca.65 cm-thick, formed during a period of relative structural stabilization that allowed use of this part of the site for habitational purposes—as documented by a circular hearth feature with a diameter of ca.1.5 m whose archeological content and enveloping sediment were differentiated as layer 21 (Fig 2, panel 3). The edges of two other, subsidence-deformed features of similar size that extend outwards, beyond the extant wall of the trench, were excavated in underlying layer 22 (Fig 4). The base of these features defined the boundary with the upper unit of the Lower Ensemble, layer 23. Layer 20 is marked by the resumption of roof-collapse events, including the fall of a massive, 2 m-long, 50 cm-wide, 60 cm-thick erosional blade that deformed the layer 21 hearth.

#### Access Corridor Upper Ensemble (layers 15–19)

Silty loam with a variable clay component, ca.1.3 m-thick, filling the space between large boulders. These boulders signal a phase of major structural destabilization with detachment of multi-ton rock masses whose bases defined the boundaries between layers 18 and 19 and between layers 19 and 20—the latter also marked by a carbonate crust, almost continuous but degraded by post-depositional phosphatization. In the reference section for the Access Corridor, this ensemble is capped by a stalagmitic crust, and discontinuous carbonate incrustations, sometimes forming thin but discrete crusts, were also observed at the corresponding elevation (top of layer 15) in the grid units leading to the 27-S Chamber. Across most of this ensemble, the characteristics of the sediment suggest that its archeological content relates to in-wash, through low-energy run-off, or overland flow, of material abandoned in the context of occupations taking place a few meters outward, in the cave’s porch—even though the largely horizontal disposition of layer 15 and its higher clay content suggest accumulation in a phase of relative stability and a decrease in the contribution of derived material. The upper units of the Access Corridor Upper Ensemble spill onto the 27-S Chamber: layer 17 abuts the steeply inclined rock face bridging it with the Access Corridor; layer 16 extended laterally to fill the space between the protruding ridges of the 27-S Chamber’s Karren-like bedrock, thusly forming the relatively regular floor upon which layer 15 came to lie.

#### Basal Cave Interior (layers 13–14)

The Access Corridor Upper Ensemble is capped by a >20-ton, ca.3 m-thick chunk of the roof fallen on grid units O-P/12-15 and obstructing communication with the outside. The silty loam filling-up the space behind this large rock mass was subdivided at the time of excavation into two layers with largely horizontal upper and lower boundaries that form the main body of the 27-S Chamber deposit: layers 13 and 14. The latter is characterized by a finer texture, with a higher clay content and a more intense reddish color. As shown by the presence of a hearth feature and corroborated by stone tool refitting [44], the archeological finds made in these two layers relate to in situ human occupation of the 27-S Chamber and adjacent areas of the Access Corridor.

#### Middle Cave Interior (layers 9–12)

As the 27-S Chamber filled-up, the cave’s interior space became more constrained and mostly functioned as a hyena den, with the remains of limited human occupation being found in the Side Passage and adjacent areas of the Access Corridor. The boundary with layer 13, marked by a stone plane and an increase in the sand and clast components of the deposit, is clear. Detailed descriptions of these units can be found in [40].

#### Upper Cave Interior (layers 7–8)

Units burying the O-P/12-15 rock mass, eventually bringing about the colmatage of the cave’s interior space and limiting human occupation to the Exterior section of the site. Layer 7 is archeologically sterile, while the artefacts and faunal remains retrieved in layer 8 largely correspond to an inward, gravity-displaced tail of the original distribution.

#### Colmatage breccia

Large boulders, éboulis and matrix forming a heavily cemented breccia that clogged the cave’s porch and the space behind as far inward as the Side Passage. The lower boundary of this ensemble is given by a well-developed, very well cemented carbonate flowstone, with clear laminar structure and a maximum thickness of 13 cm. This flowstone sealed the archeological sequence across the Access Corridor and the Side Passage, and, inward of the colmatage breccia’s rim, underlay massive stalagmitic columns that blocked the access to the 27-S Chamber and largely obstructed the Side Passage’s communication with the deeper reaches of the system.

Through the sequence, but especially in the Access Corridor, the “Swiss cheese” nature of the karst network provided all along for the existence of interstitial, void spaces underneath and to the sides of the sediment column. Therefore, subsidence adjustments, and the attendant generation of cracks and fissures, must have been recurrent through the accumulation, especially along the contact with the walls but sometimes also within the sedimentary body itself. In addition, structural layout activity was a permanent factor of instability, one that led to the frequent collapse of large rock masses fallen from the roof, on one hand, and to the dislodgement of huge chunks of the cave wall sliding along the fault’s plane, on the other. Finally, cementation by carbonate precipitation induced significant lateral variation within individual units, either layers or ensembles. In the Access Corridor, differential sediment induration was most apparent in the Lower Ensemble and the Middle Ensemble. Across these units, the areas adjacent to the wall in column N tended to be brecciated, while those adjacent to the opposite wall in column P were looser and often bioturbated.

An important implication of the complex topography of the encasing bedrock is the stepped nature of the sedimentary accumulation’s baseline. Because of this, the lowermost reaches of the sedimentary fill of the 27-S Chamber and the Side Passage remained open for long periods. Indeed, the interstitial spaces of their irregular, Karren-like floors often feature variably large, cemented remnants of the fluvial sands and silts accumulated when the Almonda flowed out through Gruta da Oliveira. These remnants are overlain by a stony cave earth accumulated during an interval that we can only very loosely constrain: between the time when the subterranean watercourse migrated downwards and the time when the accumulation of sediments in the Access Corridor reached the plane defined in those adjacent, higher-up sections by the ridges of their Karren-like bedrocks. Such intra-Karren cave earth fills are therefore susceptible of containing remains related to the lower-down occupation of the Access Corridor discarded through the accumulation of the latter’s Lower, Middle, Upper and Basal Cave Interior ensembles. To account for this possibility, the lateral equivalents of layers 16 and 13 of the Access Corridor have been differentiated as, respectively, layer 16bis in the 27-S Chamber, and layer 13bis in the Side Passage. Likewise, the deposit overlying layer 13 of the 27-S Chamber corresponds to the open, bioturbated, inward tail of the Middle (layers 9–12) and Upper (layers 7–8) Cave Interior ensembles and, accordingly, has been differentiated as layer 12bis.

### The research questions

**Fig 5:**
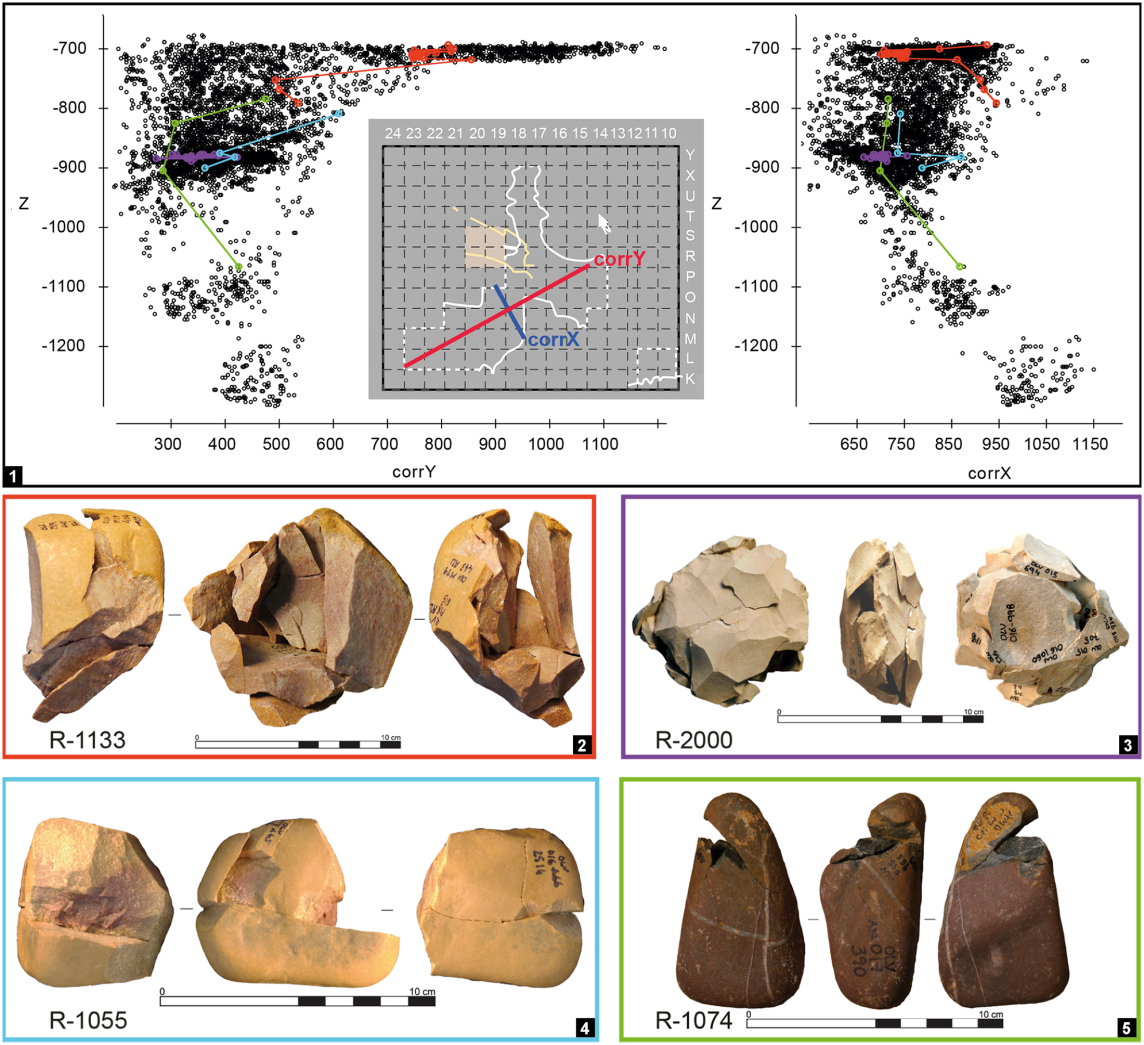
Gruta da Oliveira: Typical 3D dispersion patterns as exemplified by four refitting sets. **1**. projection of the items in each of the color-coded refit set against the vertical z-axis and the corrected horizontal axis (corrY, left; corrX, right); the position of the corrected horizontal axes used relative to the excavation grid is given in the middle. **2–5**. the refit sets in the projections. Each set is identified by a single color, used for the dots in the projections and the boxes bounding their photos. The cloud of black dots in the background represents all of the site’s piece-plotted artefacts. The R- prefix denotes the identification number of individual refit sets. Elevations are in cm below site datum.

The site formation process inferred from the stratigraphic observations made at the time of excavation and from the geoarcheological analysis of the succession begged several questions requiring a lithic taphonomy study. For instance, the preservation of fire features in layers 21 and 22 implied that the associated finds be largely in situ, in both the geological and the archeological senses, and this was indeed corroborated by the successful refit of 18 pieces documenting the reduction of a Levallois flint core in layer 21, grid units O/15-16, (refit set R-2000; Fig 5, panel 5) [61]. The inference remained to be tested, however, for the other units of the Access Corridor Middle Ensemble, layers 20 and 22.

Conversely, the mode of deposition of the Access Corridor Lower and Upper ensembles implied that a significant proportion of their archeological content be derived, along the talus of an inward-dipping slope, from occupations taking place farther out. Notwithstanding, observations made at excavation showed that intra-level refits existed, and the presence of articulated faunal remains (e.g., several elements of a lion’s paw at the interface between layers 19 and 20) supported that post-depositional disturbance be limited. Thus, these field observations remained consistent with two alternative modes of accumulation: (a) each of the individual layers differentiated in the Access Corridor’s Lower and Upper ensembles during excavation could correspond to the re-deposition of a single lens made up of items originally discarded in the context of occupations taking place in the site’s Exterior section; or (b) the integrity of such original occupations could have been lost, or significantly compromised, along the re-deposition path, in which case each of those individual excavation layers could potentially contain items that, in the primary locus of deposition—the Exterior area of the site—had been part of stratigraphically distinct lenses. Finding out which of these alternatives was the correct one required a systematic investigation of potential inter-level links. For these ensembles, a related question was whether the upper units of each, layers 23 and 15 (which represented phases of stabilization at the end of sedimentary accumulation processes leading to the complete burial of major roof-collapsed rock masses), could feature levels of assemblage integrity akin to layer 21’s.

Given the relatively imprecise boundaries of the chronometric results available for layers 15–27, which remained consistent with deposition intervals ranging between a minimum of 7000 (94–101 ka) and a maximum of 45,000 (62–107 ka) years [43], these questions had ancillary chrono-stratigraphic implications. For instance, cleavers and bifaces were found in the Access Corridor Upper Ensemble (layers 15–19), but were entirely absent both above and below [42]. Finding that each of those Upper Ensemble layers stood for a stratigraphic unit sensu stricto could lend support to the notion that this part of the archeo-stratigraphic sequence represented a significant amount of time, spanning several climate-driven depositional cycles; hence, its duration could have been closer to the maximum allowed by the chronometric results and the cleaver-plus-biface episode could have been relatively long-lived. Finding the opposite would in turn support a short duration for the archeo-stratigraphic sequence as much as for that characteristic lithic industry.

Another question that needed to be addressed was the nature and genesis of the Passage of the Column and Mousterian Cone infillings (layers 26–27). Previous interpretations oscillated between the original view that they were largely formed of vertically displaced material [52] and the hypothesis that they mostly represented material horizontally derived (washed in) from occupations emplaced outward [42]—a combination of both processes being also conceivable. Sorting out these alternatives required the inclusion of layers 26–27 in the overall refitting study, and the more so because the back end of the sedimentary column above opened to an empty space providing direct, unimpeded communication with the Mousterian Cone—the narrow fault running obliquely along units P-S/18-19 of the grid (Fig 2, panel 3).

### The approach

Our study was carried out in the premises of UNIARQ–*Centro de Arqueologia da Universidade de Lisboa* (Alameda da Universidade, 1600–214 Lisboa, Portugal), the host institution of the project to investigate the archeology of the Almonda karst system and where the Gruta da Oliveira finds are in storage. The project is carried out under excavation permits issued by the relevant authorities of the Government of Portugal (over the years: IPPAR–*Instituto Português do Património Arquitectónico e Arqueológico*; IPA–*Instituto Português de Arqueologia*; DGPC–*Direcção-Geral do Património Cultural*; excavation director, João Zilhão). The ongoing study of the finds is carried out under the 2015–2018 research project “ARQEVO–*Arqueologia e Evolução dos Primeiros Humanos na Fachada Atlântica da Península Ibérica*” (principal investigator, João Zilhão), DGPC-approved under its 2015 call for *Planos de Investigação Plurianual em Arqueologia*.

To investigate the issues above we focused on the quartzite component of the stone tool assemblage. This choice was dictated by several reasons:

- A significant proportion of the flints presents a whitish patina masking variation in color and surface appearance and rendering the identification of individual blocks more difficult than in the case of the quartzite items because these, when patinated, are only slightly so, and, in addition, are highly variable in color, texture and cortex.
- Based on the raw-material procurement study carried out for layer 14 [59], the main flint varieties used come from at least 15 km away (even though closer-by, <5 km away sources were also used), while quartzite is locally available within a radius of few hundreds of meters. The probability that complete reduction sequences could be represented in the lithic assemblages was therefore significantly higher for quartzite than for flint.
- Quartz is also locally available, and was widely used at Gruta da Oliveira. However, this raw-material is less suited for refitting studies because of its homogeneity in appearance and frequent failure to follow the principles of conchoidal breakage. In addition, the use of power tools in the excavation of the indurated parts of the deposit entailed accidental breaks, the recent surfaces of which are more difficult to identify with quartz than with flint or quartzite.

Excluding hammerstones and unmodified manuports, the total number of quartzite items catalogued in layers 15–27 is 3541. Since the inclusion of chippage and small flakes would not have been cost-effective, our study was restricted to cores, flakes, tools and thermoclasts >2 cm. In the field inventory, the count for these categories among piece-plotted items is 1718, which, pending completion of the collections’ technological study, provides a good estimate of the size of the sample targeted by our refitting work (Table 1). Once individually labelled, the finds were laid out on large tables, separated by stratigraphic provenience and sorted according to descriptive criteria (size of quartz grains, color, sub-cortical alteration). After an initial phase of intra-level analysis, links with adjacent units were searched for; eventually, the search was extended to non-adjacent units.

**Table 1:** Refit success rate (quartzite). Variation in the percentage of refitted items (debitage, debris, and thermoclasts) per stratigraphic unit of provenience.

Layer	Refitted (N)	Total*		Piece-plotted**	
		N	Refitted	N	Refitted
**15**	83	993	8%	421	20%
**16**	26	253	10%	148	18%
**16bis**	7	55	13%	23	30%
**17**	57	307	19%	165	35%
**18**	78	487	16%	234	33%
**19**	94	433	22%	215	44%
**20**	63	336	19%	209	30%
**21**	15	120	13%	58	26%
**22**	45	190	24%	109	41%
**23**	15	53	28%	36	42%
**24**	4	30	13%	16	25%
**25**	1	6	17%	2	50%
**26**	22	138	16%	46	48%
**27**	15	140	11%	36	42%
**TOTAL**	**525**	**3541**	**15%**	**1718**	**31%**

* cobbles, hammerstones and other unmodified manuports excluded

** used as a proxy for the larger-sized material targeted by the refitting study

For the assessment of assemblage integrity and post-depositional disturbance, refit sets linking items with more than one stratigraphic unit of provenience were assigned to a “layer of original production” (LoP) (Table 2, S1 and S2 Tables; Fig 5). This assignment was based on the three-dimensional distribution of the sets’ components and the following assumptions, derived from field observations, the geoarcheological data, and the results of the refitting study previously carried out for layers 8–14:

- As a rule, post-depositional vertical displacement occurred downwards rather than upwards.
- For any given unit, items retrieved in the syn-depositionally indurated areas of the Access Corridor (broadly, columns N and O) are more likely to be in situ than items retrieved in the looser, potentially bioturbated areas of the same unit (broadly, column P).
- Items retrieved in burrowed or root-disturbed areas explicitly identified as such in the field documents as well as items found adjacent to the cave wall (in column P) or to the steeply inclined bedrock plane bridging the Access Corridor and the 27-S Chamber (i.e., depending on stratigraphic depth, in rows 18–19 or 17–18) are most susceptible to represent long-distance displacement and, hence, contamination of a given unit’s assemblage by material intruded from above.

**Table 2:** Gruta da Oliveira typical refit sets. Classification and interpretation after the nature of stratigraphic links and rationale for layer of production (LoP) assignment.

Refit #	Archeo-stratigraphic unit of provenience for items in the refit set														Total	LoP	Nature of links	Interpretation
	15	16	16 bis	17	18	19	20	21	22	23	24	25	26	27				
1055	–	–	–	–	1	–	2	1	–	–	–	–	–	–	4	20	Adjacent	The layer 18 item was found in a bedrock fissure in N18; it was labelled "layer 18" in the field based on elevation criteria. This item predates the accumulation of layer 18 and is an instance at lower elevation of the problems generated by the open nature of the sediment filling interstitial spaces of the 27-S Chamber’s Karren-like bedrock that underpin the differentiation of layer 16bis (see text).
1074	–	–	–	1	–	1	–	–	–	1	–	–	1	–	4	?	Anomalous	The layer 17 item comes from square O17, close to the wall but in a brecciated area, and represents the parsimonious LoP for this set. However, the distribution’s scatter does not allow secure inference and no LoP is assigned.
1133	19	1	–	1	1	–	–	–	–	–	–	–	–	–	22	15	Long	The items from layers 16, 17 and 18 come from square P18 and are downwardly displaced along the cave wall. The layer 15 items all come from the 27-S Chamber. This refit set is a good example of the processes affecting the stone tool assemblage (broad stratigraphic integrity coupled with some vertical displacement between adjacent levels and occasional long-distance links provided for by wall effects and bioturbation).
2000	–	–	–	–	–	–	2	16	–	–	–	–	–	–	18	21	Intra	The layer 20 items are from the the interface with layer 21 and reflect *décapage* error, not post-depositional displacement (see text).

In some cases, no LoP could be assigned, namely when the refitting set was composed of only two items coming from either potentially disturbed areas or layers 26–27. More rarely, assignment was impossible because the distribution spanned non-adjacent units and the direction of the displacement could not be ascertained.

The refit sets for which LoP’s could be proposed were then classified into categories based on the nature of the links, defined according to the stratigraphic distance encompassed: **intra**, when only items from a single layer were found in the set; **adjacent**, when items from an immediately underlying or overlying layer were included; **long**, when the links extended beyond the units adjacent to the assigned LoP. These categories were considered “normal” as the distributions respected stratigraphy within site formation and excavation constraints: on one hand, links with units adjacent to the assigned LoP are to be expected as a result of surface dynamics, bioturbation, and excavation error; on the other hand, all links beyond adjacent units in sets for which an LoP nonetheless could be assigned concerned items found within pockets of localized post-depositional disturbance (e.g., burrows), along the encasing bedrock in areas affected by wall effects (e.g., column P), in the Mousterian Cone/Passage of the Column, or in the fill of the 27-S Chamber’s basal Karren (layer 16bis). The refitting sets with inter-layer links that could not be explained per these factors and for which no LoP could be assigned were considered anomalous (“non-normal”).

The rationale for considering as “normal” the sets in the **adjacent** category of refit links derives from the diffuse nature of most stratigraphic interfaces, compounded by the difficulty encountered in indurated areas when trying to follow them. A case in point concerns the individualization of layer 21. The plane defined by the black staining found at the base of spit A66, denoting the upcoming emergence of a large hearth (Fig 2, panel 3), was defined as the lower boundary of layer 20. Layer 21 was in turn defined as the 5–10 cm-thick slice of the deposit excavated as spit A67 down to the base of that feature. In the periphery of the latter, especially in column N, however, the induration of the sediment often requested the power tool-assisted removal of chunks that sometimes were themselves as thick as, or thicker than A67 itself. Consequently, for finds made at this elevation, a measure of error is expected to exist in the spit-to-layer correlation and, hence, in the stratigraphic assignment made in the field (e.g., to layer 21 instead of layer 20, or to layer 22 instead of layer 21, and vice-versa). Refit links bridging these layers are therefore likely to reflect such kinds of “excavation error” rather than post-depositional disturbance—which, given the very preservation of the layer 21 hearth feature, can only have been minimal.

Likewise, limited exchange of items across the interface between two adjacent layers is to be expected due to trampling, small-scale burrowing, and progradation (the latter given the position of the Access Corridor trench, at the back end of the inward sloping connection with the Exterior area). Such interface porosity introduces a measure of fuzziness in the definition of assemblages, but is of no consequence for the overall typological and technological assessment of the material retrieved within the thick sediment packages found above and below the boundaries of defined stratigraphic units. The same logic applies to the cases when a refit set links a given LoP with items displaced downward through wall effect, even if as far down as the Mousterian Cone/Passage of the Column—the sets whose refit links fall in the **long** category. Such connections document loss to erosion and the contamination of underlying units of the sequence, but are of no consequence for the assessment of the stratigraphic integrity of the displaced items’ layer of origin.

Bearing in mind these data and premises, we analyzed the stratigraphic integrity of individual units of the sequence in light of: (a) the projection of linked items along the axis of deposition, which is oblique to the axis of the grid and so required prior transformation of the (x,y) field coordinates into corrected ones (corrX, corrY) (for the sake of simplicity, in these projections the material from layer 16bis has been considered together with layer 16); (b) the tabulation of the proportion of sets with **intra** and **adjacent** refit links within individual LoP’s; and (c) the tabulation of the same proportions for the ensembles of production (EoP) into which the analysis of (a) and (b) led us to group the different archeo-stratigraphic units.

## Results

The refitted sets are 150, with the most complete comprising 22 objects. R-2000 is a flint refit, the other 149 are all quartzite. The variation across the sequence in the frequency of the different categories of refit sets is given in Tables 3 and 4. In Table 3, the data are presented per individual LoP. In Table 4, the same data are organized into the analytical ensembles judged to be those best suited for the organization of the collections. With regards to the research questions that we targeted, these data support several conclusions.

**Table 3:** The different types of refit sets. Distribution per layer of production (LoP).

LoP	sets (N)	Nature of stratigraphic links (N)				Intra+adjacent	Anomalous
		Intra	Adjacent	Long	Anomalous		
**15**	28	16	3	9	–	68%	–
**16**	6	0	1	5	–	17%	–
**17**	10	4	2	4	–	60%	–
**18**	23	6	8	9	–	61%	–
**19**	25	8	11	6	–	76%	–
**20**	14	4	4	6	–	57%	–
**21**	5	3	1	1	–	80%	–
**22**	15	6	2	7	–	53%	–
**23**	2	–	–	2	–	0%	–
**24**	–	–	–	–	–	–	–
**25**	–	–	–	–	–	–	–
**?**	22	2	5	0	15	32%	68%
**TOTAL**	**150**	**49**	**37**	**49**	**15**	**57%**	**10%**

**Table 4:** The different types of refit sets. Distribution per ensemble of production (EoP).

EoP	sets (N)	Nature of stratigraphic links (N)				Intra+adjacent	Anomalous
		Intra	Adjacent	Long	Anomalous		
**15**	28	16	7	5	0	82%	–
**16–19**	74	42	16	16	0	78%	–
**20**	14	4	6	4	0	71%	–
**21–22**	21	11	7	3	0	86%	–
**23–25**	3	0	3	0	0	100%	–
**?**	10	2	5	0	3	70%	30%
**TOTAL**	**150**	**75**	**44**	**28**	**3**	**79%**	**2%**

### Genesis of the Mousterian Cone/Passage of the Column

**Fig 6:**
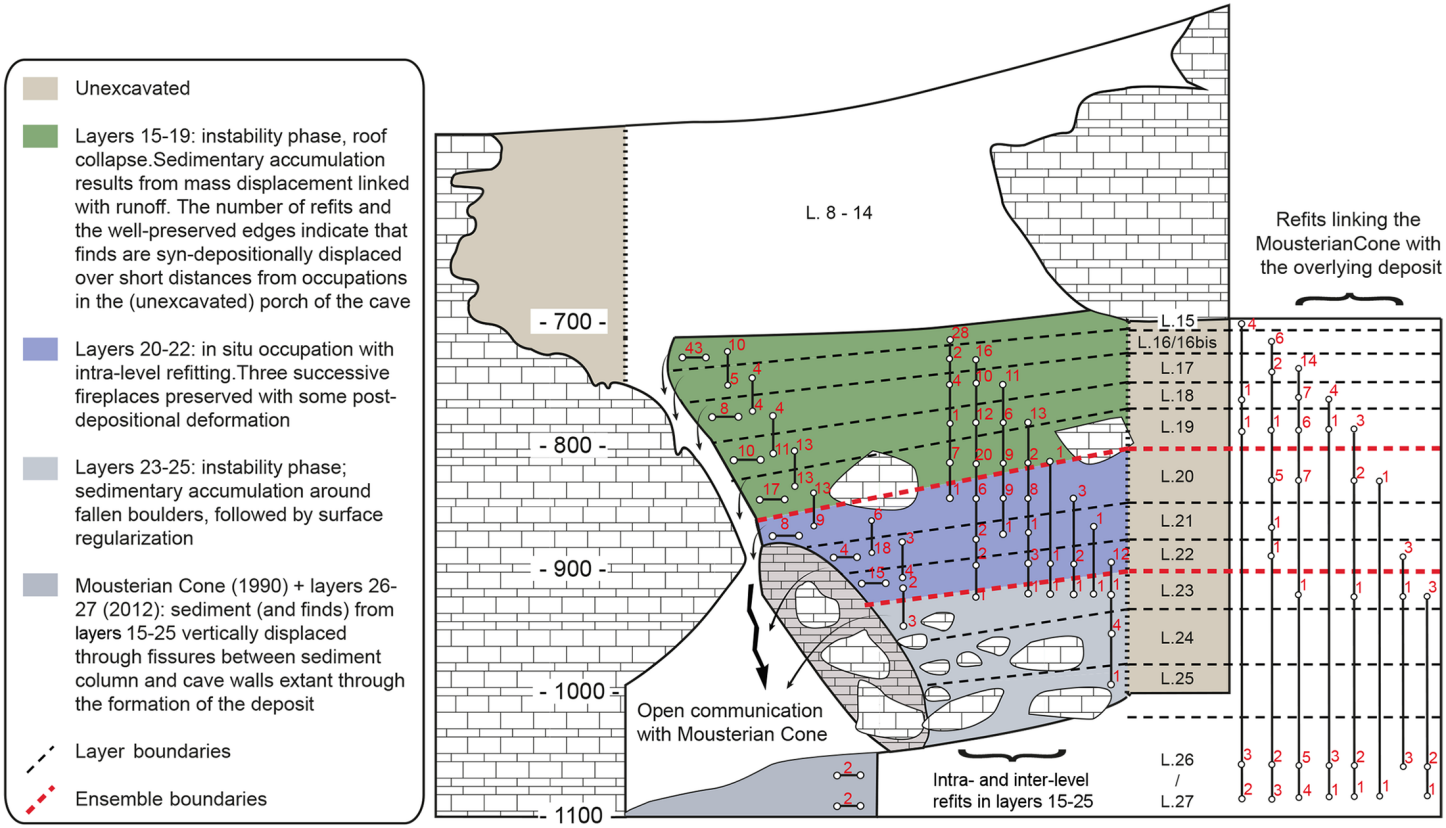
Gruta da Oliveira: Distribution of refit links across layers 15–27 projected on a schematic rendition of the Access Corridor’s stratigraphic sequence. The vertical black lines denote inter-level refits, the horizontal black lines denote intra-level refits. The red numbers associated with the vertical lines denote the accumulated number of instances where items from a given layer refit with items in each of the underlying layers. The refits with the Mousterian Cone documenting long-distance displacement are plotted separately in similar manner. Elevations are in cm below site datum.

We find that several long-distance refits link layers 26–27 with all other units of the overlying sequence. In addition, only two intra-level refits were made within this ensemble, each comprised of no more than two items (Fig 6). This evidence shows that the stone tools found herein do not form a coherent assemblage. They represent syn- or post-depositional, downward displacement of items whose LoP spans the entire sequence targeted by our study, explaining the high refit success rate obtained—measured against the totals for piece-plotted items, among the highest in the sequence (Table 1).

These items’ displacement process may have consisted of (a) gradual, attritional, intermittent “drizzling” of artefacts along the voids between the sediment column and the cave walls, (b) mass displacements of chunks of the sedimentary fill in the context of such catastrophic events as those underpinning the accumulation of the multi-ton boulders lying on the surface of layers 20, 19, and 15, or (c) a combination of both. The two intra-level refits suggest the displacement of finds with maintenance of original connections, which supports (b) or (c)—as does the fact that, so far, no refit links with layers 8–14 exist. With present evidence, however, the alternative cannot be resolved. The one conclusion that can be drawn for now is that layers 26–27 do not form a valid analytical unit for the study of the cave’s archeology and so their content ought to be removed from considerations relating to diachronic change in the human use of the site.

### Assemblage integrity

**Fig 7:**
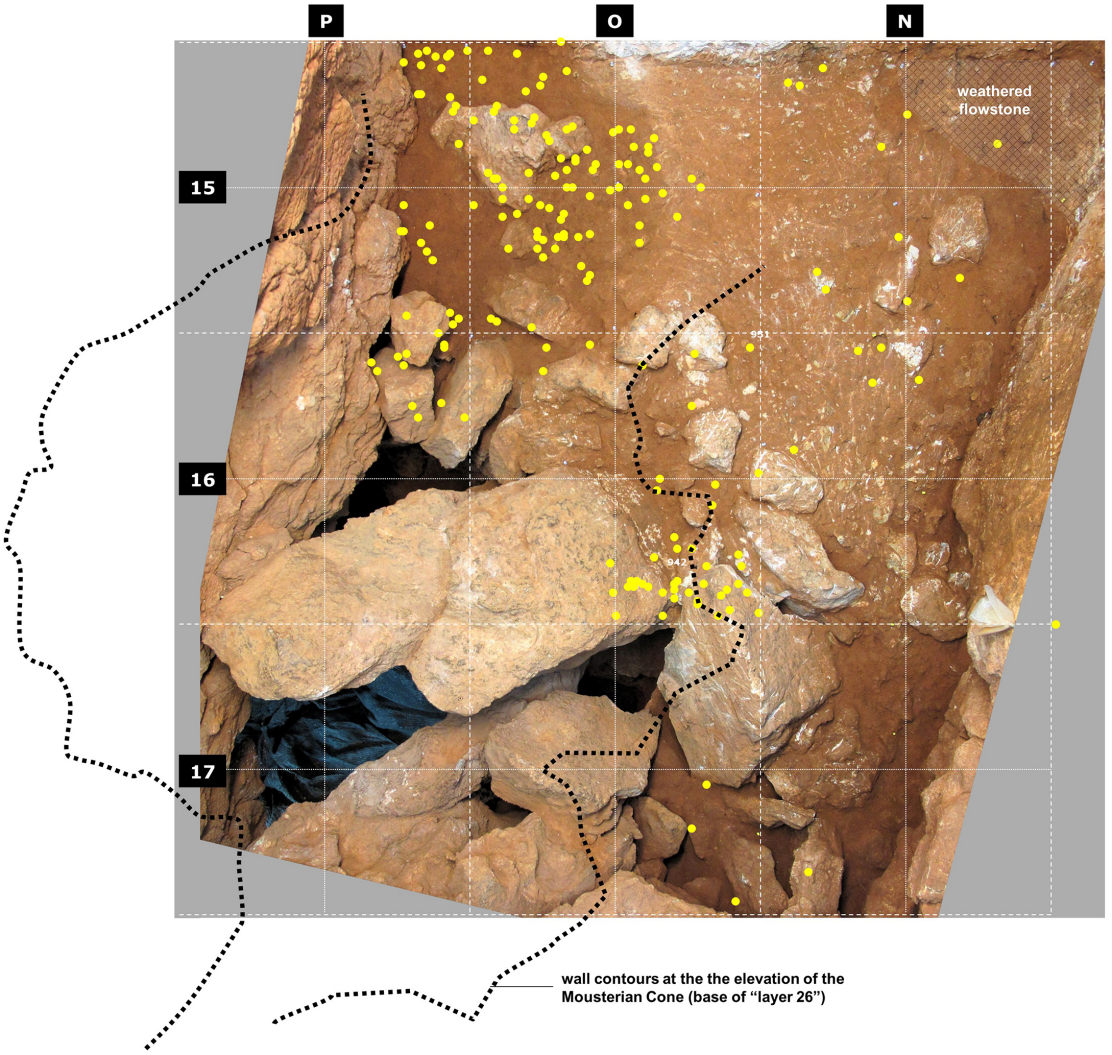
Gruta da Oliveira: Horizontal distribution of piece-plotted lithic artefacts from layer 23. The dots represent the (x,y) grid coordinates and they are plotted on an orthorectified, composite image of the interface between layers 23 and 24 (base of spit A69). Note the concentration in the subsidence-affected area above the largely openwork éboulis filling the connection between the Access Corridor and the Mousterian Cone/Passage of the Column (layers 24–25).

In layers 23–25, lithic artefacts are few and, corroborating field observations, refits primarily demonstrate links with layer 22, from where, at the time of excavation, the burnt, hearth-related bones and flints retrieved in layer 23 were inferred to derive. When the horizontal distribution of the piece-plotted items from this layer is considered (Fig 7), the pattern is easy to explain. Indeed, most come from areas that were subject to strong subsidence and attendant deformation and disturbance effects: (a) the ca.1 m-wide band along the P-column wall of the cave, which is also bioturbated (as clearly seen in the reference cross-section; Fig 4), or (b) the back end of the trench—atop the coarse, often openwork éboulis forming layers 24–25 or above the large void situated between the sedimentary column and the Mousterian Cone. The few and scattered finds made in the middle of the trench and against the opposite wall come from apparently undisturbed areas, and that is likely to be the prevalent condition in the unexcavated areas of the site outwards from the Access Corridor trench. Be it as it may, the stone tool sample from layers 23–25 available to us mostly comes from an area of the trench where the integrity of the deposit cannot be warranted. Therefore, these lithic assemblages are best deemed potentially heterogeneous and conservatively removed from further consideration in terms of human behavior.

**Fig 8:**
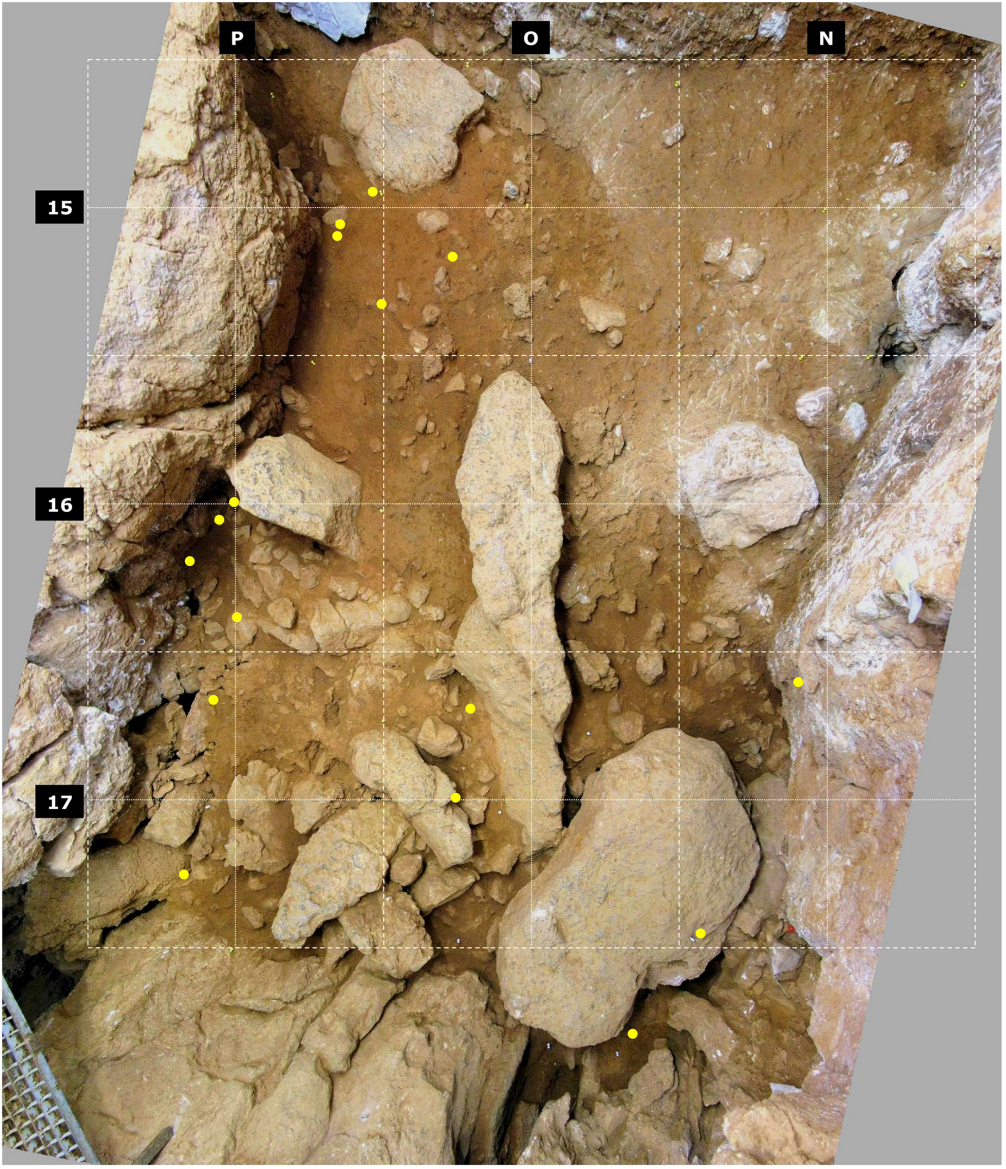
Gruta da Oliveira: Horizontal distribution of refitted lithic artefacts from layer 20 that reflect downward displacement from overlying units. The dots represent the (x,y) grid coordinates and they are plotted on an orthorectified, composite image of the base of the first spit (A65) into which layer 20 was subdivided at the time of excavation. Note the concentration in areas susceptible of strong wall effects or consisting of openwork éboulis.

Layers 21 and 22 have a strong anthropogenic component, including hearths, which is suggestive of actual human occupation of the Access Corridor—and intra-level refits are indeed numerous here, which is reflected in these units’ refit success rate (for layer 21, the highest in the sequence when only the piece-plotted items are considered; Table 1) and in the percentage of **intra** plus **adjacent** refits (86% when layers 21 and 22 are considered together as a single analytical ensemble; Table 4). Reflecting its stratigraphic interface nature, the upper part of layer 20 is linked to the stratigraphic units above by a significant number of refits, explaining why the percentage of **intra** plus **adjacent** ones is significantly lower in either the LoP or the EoP frames of reference (57% and 71%, respectively). When analyzed in detail, however, most anomalies concern items retrieved in the periphery of the distribution—against the cave walls and in the openwork éboulis at the back end of the trench (Fig 8), where the interface between layers 19 and 20 was poorly defined and, at the time of excavation, largely established on the basis of altimetric criteria (i.e., in these peripheral areas the interface plane was defined by projection from the dip observed in the intact areas, with items falling above being assigned to layer 19 and items falling below being assigned to layer 20). Excluding from the studied assemblages the items retrieved in these problematic parts of the trench therefore ought to suffice to generate a stratigraphically reliable sample.

Based on the geoarcheological evidence, the artefact component of layers 15–19 was inferred to result primarily from mass transport over a short distance, the original occupations having been located a few meters outward [40,42]. The higher proportion of **long** refits in these units (especially in layer 16; Table 3) indicates that the validity for human behavior analysis of the archeo-stratigraphic subdivisions made at the time of excavation needs to be critically assessed.

**Fig 9:**
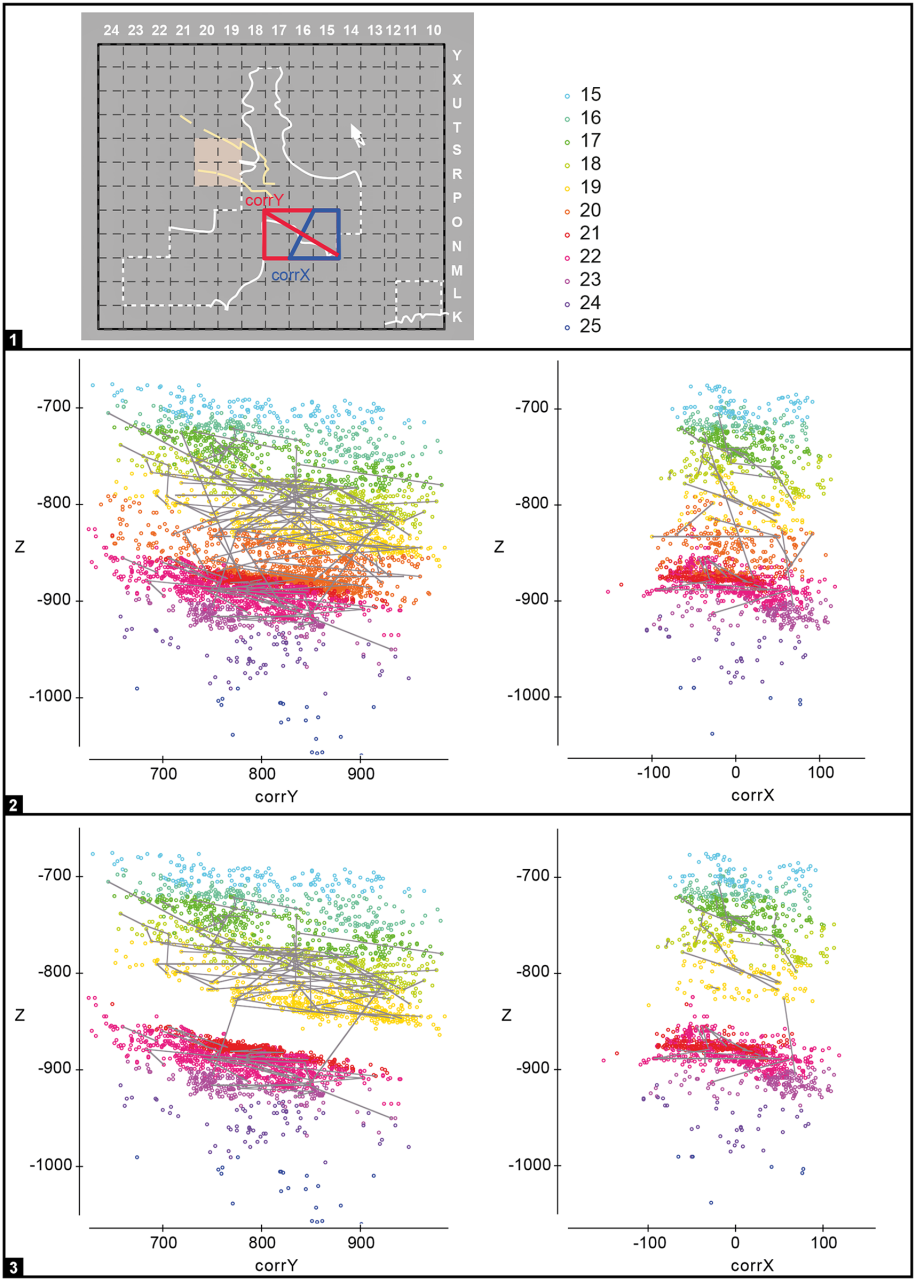
Gruta da Oliveira: Refit links indicated on the projection of piece-plotted lithic artefacts from the Access Corridor. To filter out the impact of wall effects, the plot only includes items from grid units N-O/15-17. **1**. color codes per stratigraphic unit of provenience and position of the axes used relative to the excavation grid. **2**. projection on the corrY- and z-axes (left) and on the corrX- and z-axes (right). **3**. the same projections with exclusion of the layer 20 material. Note the predominantly sub-horizontal, stratigraphically consistent orientation of refit links, with very few long-distance, inter-level connections. Removing from the plots layer 20, in which row 17 is also affected by post-depositional disturbance, makes the pattern clearer. Elevations are in cm below site datum.

Below layer 15 (i.e., for layers 16–19), the refitting patterns are consistent with inward displacement along a slope leading from the entrance to the back of the cavity (Fig 9). They also show that, if it at all existed, the original micro-stratigraphic organization into different occupation lenses was lost in the transport process. For the purposes of archeological analysis, these layers’ content should therefore be treated together as a single unit of analysis. Indeed, in the EoP frame of reference, the proportion of **intra** plus **adjacent** refit links obtained for the layers 16–19 ensemble rises to 78%, comparable to that obtained for the better-preserved parts of the sequence (Table 4). Fig 9 also illustrates well how removing from the plot for columns N and O the items for which layer 20 is the LoP (to additionally filter out the impact of the disturbance displayed by this layer in row 17; Fig 8) makes the separation between the Upper Ensemble (layers 15–19) and the Middle Ensemble (layers 20–22) much clearer: when this is done, only two refits connect them.

**Fig 10:**
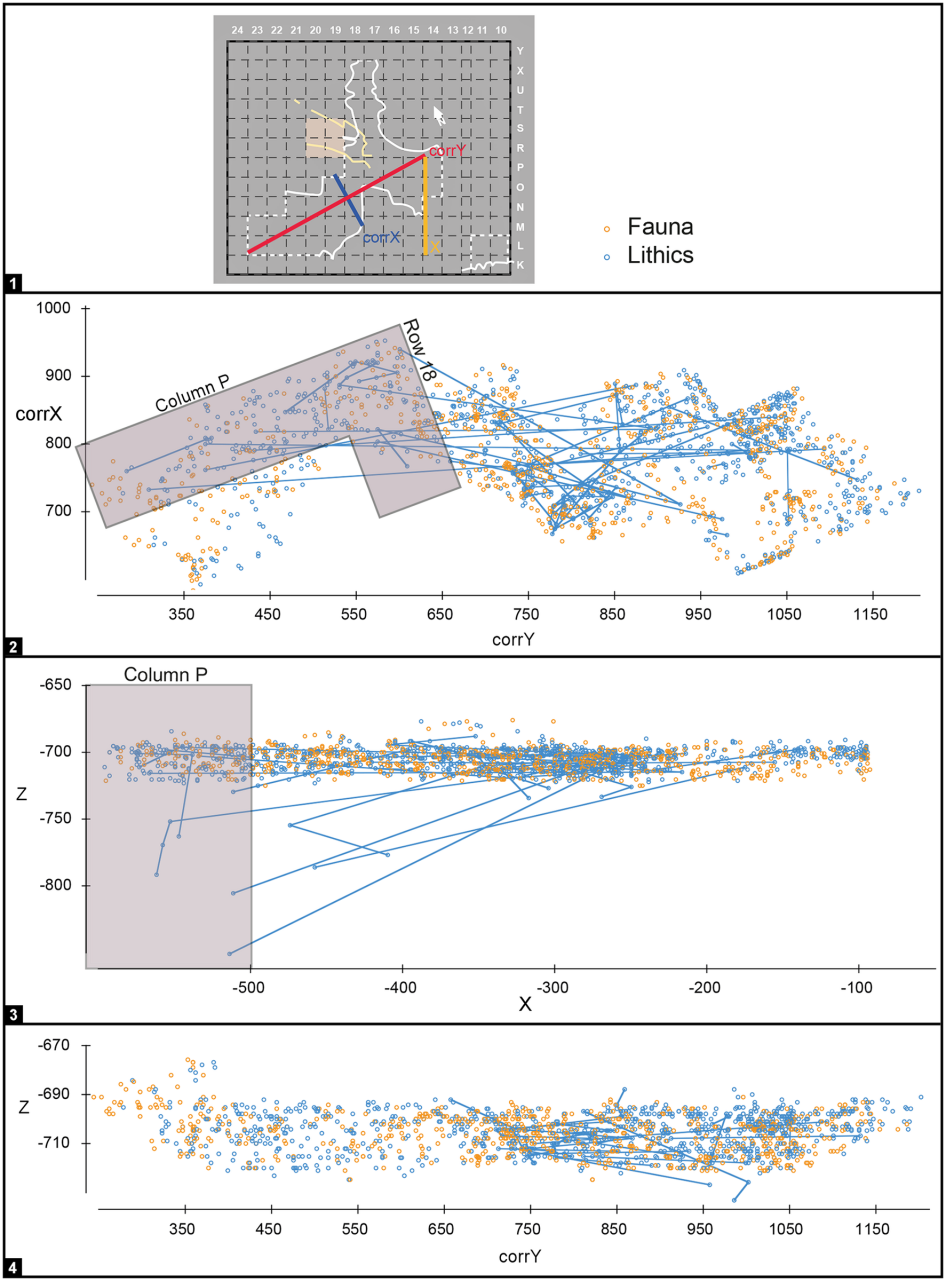
Gruta da Oliveira: Refit links of sets for which layer 15 is the LoP (layer of production). The refitted items and associated refit links are shown on projections of all the piece-plotted finds, including faunal remains, from layer 15. **1**. color codes per type of find and position of the axes used relative to the excavation grid. **2**. projection on the horizontal, corrX- vs corrY-axes. **3**. projection on the x- and z-axes, illustrating the concentration of long distance-linked items in column P of the grid. **4**. projection on the corrY- and z-axes, excluding items from column P and row 18 of the grid, illustrating a network of horizontal connections restricted to the 27-S Chamber. The finds made in layers 26–27 are excluded from the plots. Elevations are in cm below site datum.

Layer 15, however, stands out within the Upper Ensemble due to the high percentage of **intra** plus **adjacent** refit links—82%, in an EoP frame of reference (Table 4). This is consistent with the fact that layer 15 represents the last stage of the sedimentary colmatage of the space left between the large boulders forming the skeleton of the Access Corridor Upper Ensemble; at that time, the surface of the fill had become largely horizontal, and surface dynamics was reduced. Coupled with the limited vertical dispersion of the items incorporated in refit sets for which layer 15 is the LoP (Fig 10), this evidence warrants treatment of the corresponding stone tool assemblage as an independent unit of analysis. The fact that layer 15’s refit success rate is comparatively low (Table 1) does not contradict this assessment; it simply reflects the much larger size of the assemblage (twice the next largest one, that for layer 18), as it is to be expected that success rates and assemblage size will vary in inverse manner.

### Post-depositional dynamics

**Fig 11:**
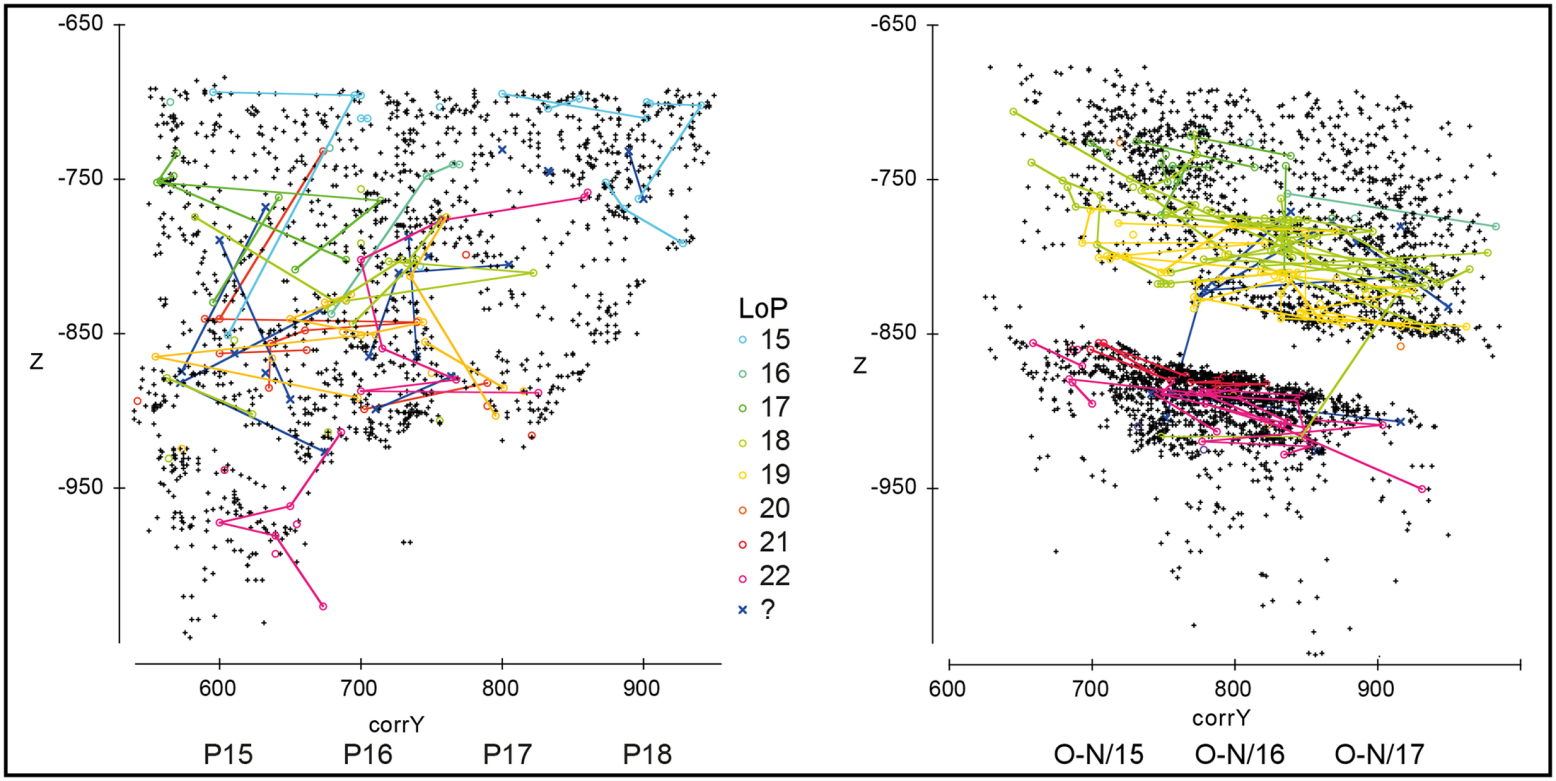
Gruta da Oliveira: Vertical distribution of piece-plotted items from the Access Corridor according to LoP (layer of production). **Left**. items in column P. **Right**. Items in columns N and O, excluding layer 20. Note the numerous long-distance links in column P in contrast with the stratigraphic consistency of the distributions elsewhere in the trench. Elevations are in cm below site datum.

The vertical projection of refitted items along the axis of deposition illustrates well the mechanisms of post-depositional disturbance at work in Gruta da Oliveira that participated in the formation of the part of the succession studied here. In Fig 11, we look into the situation in the Access Corridor and, to highlight the impact of wall effects in column P of the grid (also apparent in Fig 10), we plotted the stone tools therein separately from those in columns N and O. The refit lines form a pattern of relatively well-preserved sub-horizontal accumulations in the latter, except with regards to layers 16–19, in which the observed dip is more important. This exception agrees with the mode of formation inferred from the geoarcheological data, which would have favored a telescoping of the deposit inward of the original point of accumulation.

**Fig 12:**
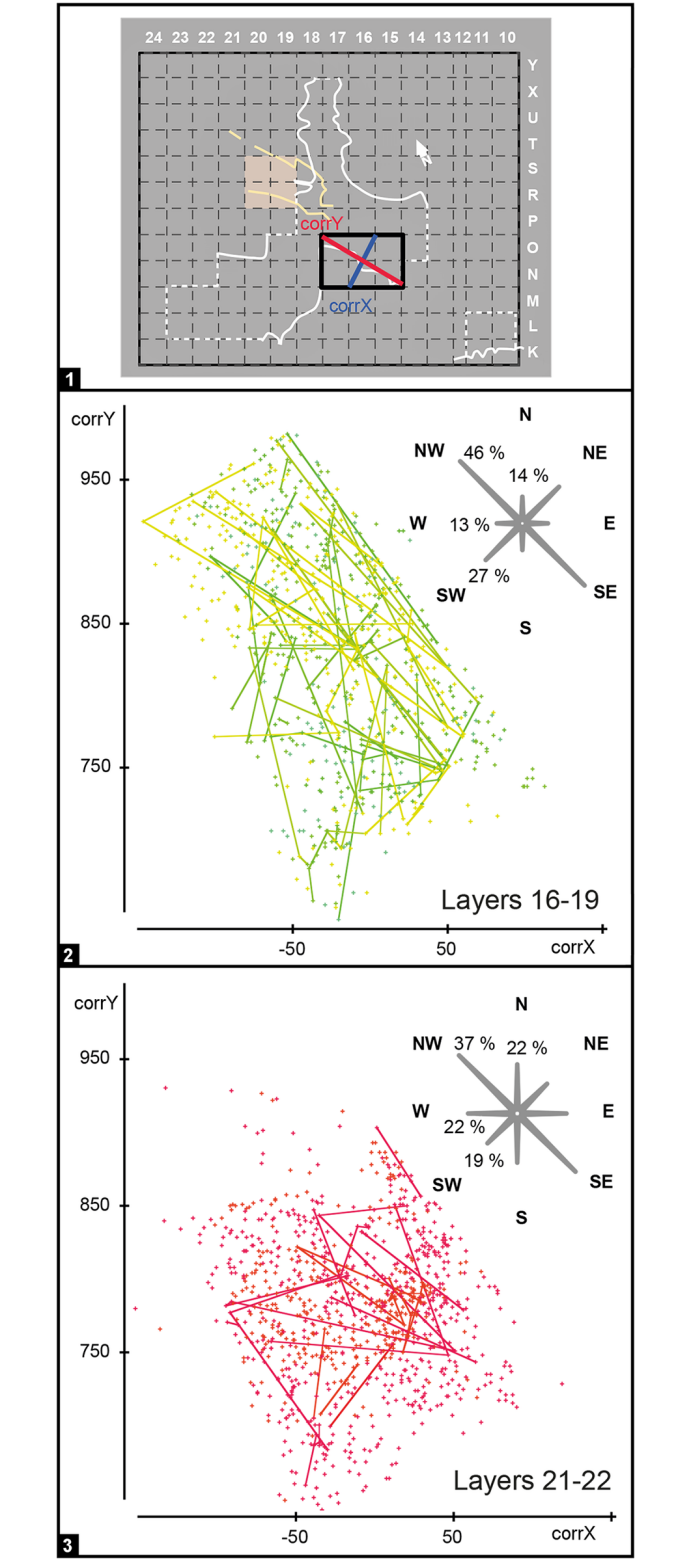
Gruta da Oliveira: Horizontal distribution of piece-plotted items from grid units N-O/15-17 and orientation of the refit links. 1. position of the axes used relative to the excavation grid. **2**. projection on the horizontal, corrX- vs corrY-axes of artefacts included in refit sets whose assigned LoP lies in layers 16 to 19, and radar graph showing the orientation of the corresponding refit links. **3**. projection on the horizontal, corrX- vs corrY-axes of artefacts included in refit sets whose assigned LoP lies in layers 21–22, and radar graph showing the orientation of the corresponding refit links.

The horizontal projection of refit links for layers 16–19 and 21–22 (Fig 12) adds support to the inferences on the dynamics of sediment accumulation based on the geoarcheological data. For layers 16–19, the preferential direction (NW-SE) of the links is clearly apparent, and many connections exceed 1 m. For layers 21–22, the orientation of the links is more homogenous and most connection distances are under 1 m. These observations support that the layers 21–22 pattern derive from human activity-induced, syn-depositional scatter of debris related to the occupation of the Access Corridor itself. The preferentially oriented, longer distance connections seen in the case of layers 16–19 are in turn consistent with post-depositional displacement into the Access Corridor of debris related to occupational activities taking place in the Exterior area of the site.

The horizontal distribution of all the items that go into refits for which layer 15 is the LoP (column P and rows 17–18 included; Fig 10) shows a concentration in the 27-S Chamber. This distribution suggests that the latter was the actual emplacement of the human occupation, with the items retrieved in the Access Corridor primarily representing a peripheral scatter. This is corroborated by (a) the fact that several refits concern items retrieved quite nearby, over no more than 1–2 m^2^ of the 27-S Chamber, a case in point being refit R-1133, which conjoins 22 items, 14 of which from grid unit M19 (Fig 5, panel 4), and (b) the absence of refits linking items retrieved in the Access Corridor alone. The morphology of the cave wall must underpin this pattern to a significant extent, as, at this elevation, a major salient occupies most of grid unit O17 and strangulates the communication between those two sections of the cave plan. Fig 10 also highlights the long-distance vertical displacement of a few layer 15 items, while Fig 6 shows how some moved as far down as the Mousterian Cone. Detailed analysis of the projections against the field documents shows that this displacement takes place in two steps: in the first, objects syn-depositionally disperse from the 27-S Chamber to the Access Corridor; in the second, some are post-depositionally displaced downward, along the planes of contact between the sedimentary deposit and the encasing bedrock found in column P and in row 18.

### Diachronic change

**Fig 13:**
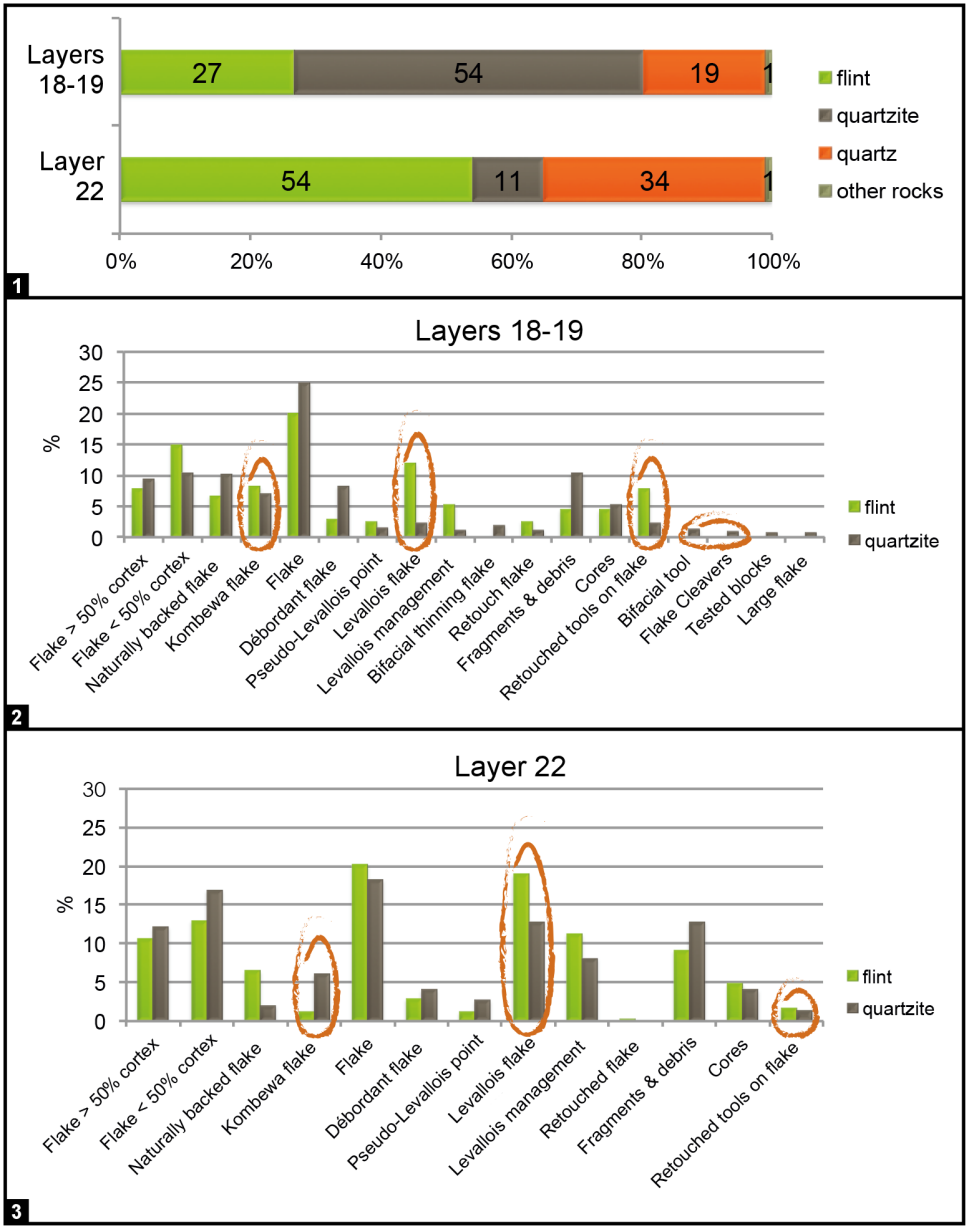
Gruta da Oliveira stone tools: Raw-material and technology. **1**. representation of the different raw-materials in the analyzed samples. **2**. distribution of technological categories per raw-material (flint and quartzite only) in the layers 18–19 sample. **3**. distribution of technological categories per raw-material (flint and quartzite only) in the layer 22 sample. The categories illustrating the main differences between the two samples are highlighted.

**Fig 14:**
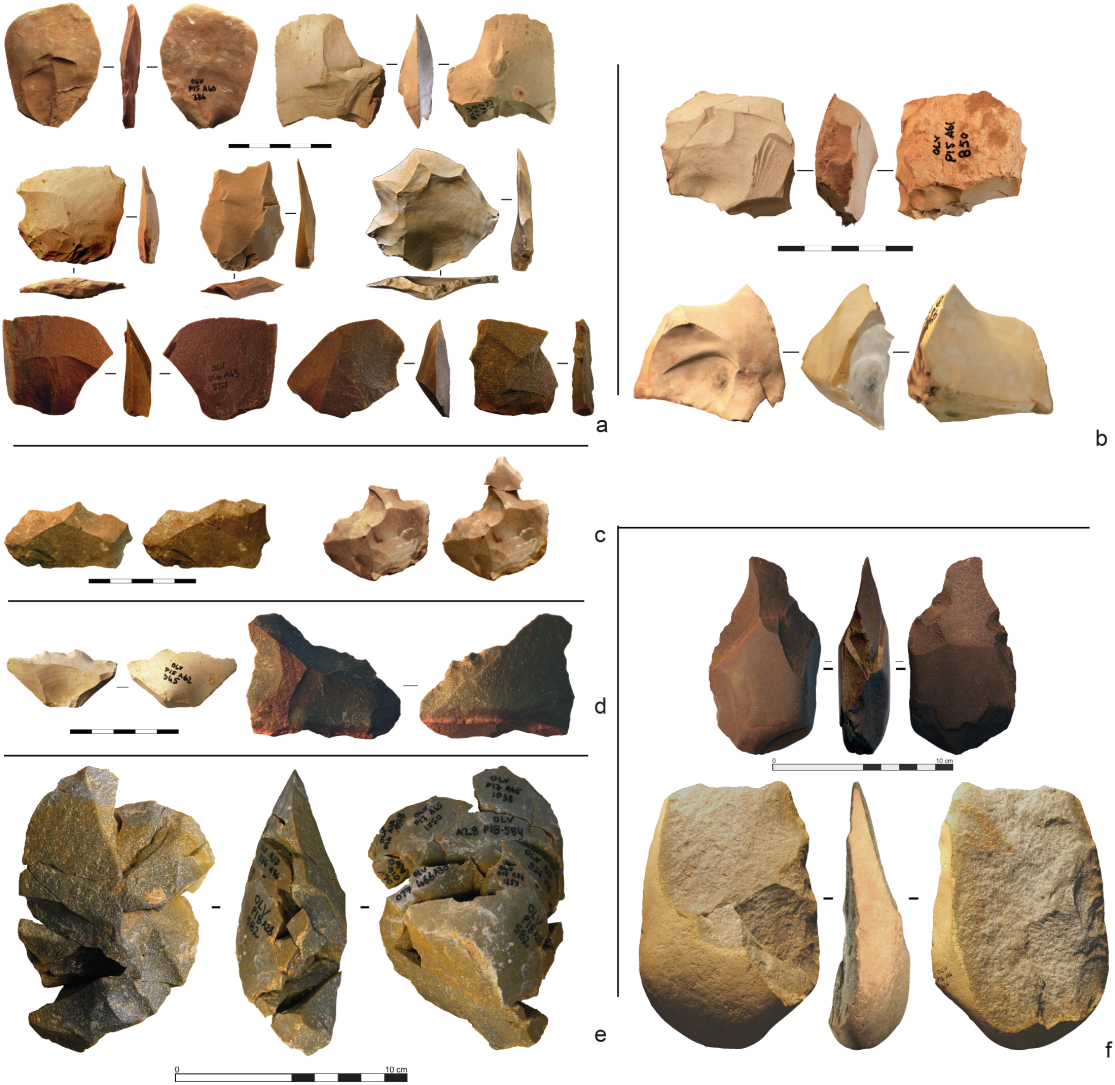
Gruta da Oliveira stone tools (layers 18–19). **a**. unretouched flakes (flint and quartzite); **b**. cores (flint); **c**. denticulates, and the same with refitted retouch byproducts (flint); **d**. denticulates (flint and quartzite); **e**. refit set R-1011, for which layer 18 is the LoP (layer of production) (quartzite); **f**. biface and flake-cleaver (quartzite).

**Fig 15:**
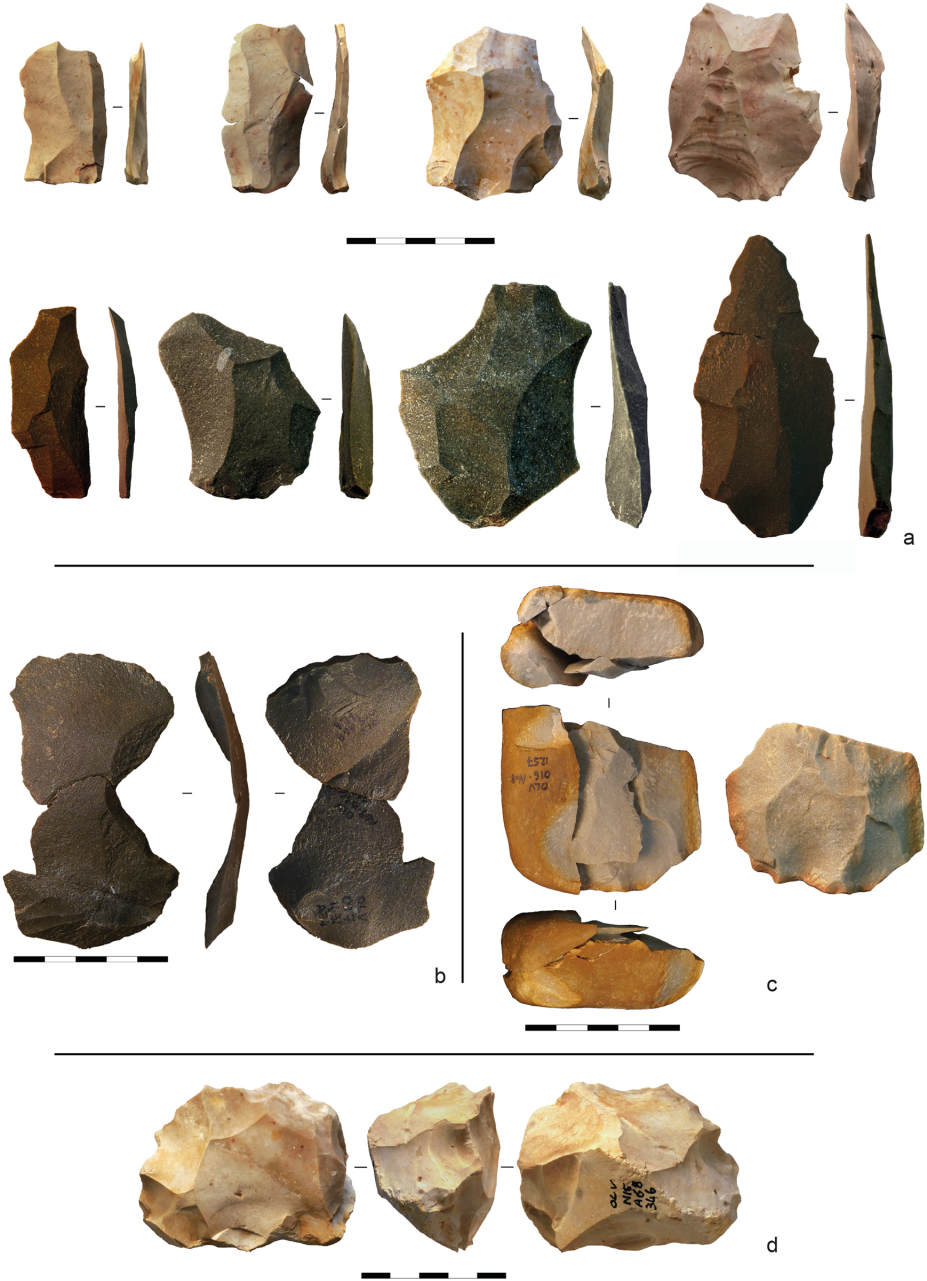
Gruta da Oliveira stone tools (layer 22). **a**. unretouched flakes (flint and quartzite); **b**. refit set R-1000, for which layer 22 is the LoP (layer of production), illustrating a Kombewa-type reduction (quartzite); **c**. refit set R-1012, for which layer 22 is the LoP (left), and a view of the core without the refitted artefacts (right) (quartzite); **d**. core (flint).

To make a preliminary assessment of change in raw-material economy, technology and typology across the studied sequence we compared the assemblage from layers 18–19 (combined) with that from layer 22. These units were chosen to minimize background noise resulting from the impact of wall effects in the composition of layers situated in intermediate position. Items <2 cm were excluded. The comparisons concern a total of 798 objects for the layers 18–19 sample and of 564 objects for the layer 22 sample (Figs 13–15).

Flint artefacts dominate in the layer 22 sample, followed by quartz and quartzite, while quartzite dominates in the layers 18–19 sample (Fig 13). Given that the samples are of sufficient size, these changes in raw-material preference must reflect differences in the use of regional resources related to site function and techno-economic choice.

The samples also differ in the type of reduction primarily used. In the layers 18–19 sample, preferential Levallois debitage is dominant on flint, while quartzite flakes are extracted following a centripetal method and only very few are Levallois; the number of Kombewa flakes in both flint and quartzite is also substantial (Fig 14a and 14b). In the layer 22 sample, flint and quartzite are reduced in similar manner; the Levallois method is clearly dominant, mostly preferential and, secondarily, recurrent unipolar (Fig 15).

Retouched tools made on flake blanks are rare in the layer 22 sample. Better represented in the layers 18–19 sample, their overall percentage remains, however, low (8% of flint artefacts). The blanks selected for retouch are ordinary or naturally-backed flakes, never Levallois ones. Tool-types are mostly denticulates and notches, followed by flakes with partial retouch, with some sharpening and re-sharpening being apparent in the layers 18–19 sample (Fig 14c and 14d). The latter sample also contains a limited number of quartzite macro-tools (flake-cleavers and bifaces; Fig 14f), which are entirely absent from underlying units and, at the site, only occur in units of the Access Corridor Upper Ensemble (layers 15–19).

In the Portuguese Middle Paleolithic, these Gruta da Oliveira Upper Ensemble levels are currently the only ones known to contain flake-cleavers and bifaces. One biface has been reported from the open-air site of Sapateira 2 (Baixo Alentejo), assigned to the Middle Paleolithic, but the archaeological finds made therein are contained in a secondary colluvium [62] and, therefore, the integrity and dating of the assemblage must be considered an open issue. At Gruta da Oliveira, refits link all the archeo-stratigraphic units in which these macro-tools are found, suggesting that the interval represented by their accumulation was rather short. The absence of such tools from over- and underlying units certainly cannot be due to issues of assemblage size, and no evidence suggests that it owes anything to functional factors. These patterns plead for the presence of flake-cleavers and bifaces in the Middle Paleolithic of Atlantic Iberia to be a regional industrial marker of wider chrono-stratigraphic significance.

## Discussion and conclusions

Our study has shown that, at Gruta da Oliveira, the use for archeological analysis of either the archeo-stratigraphic units differentiated at the time of excavation or the geo-stratigraphic ensembles defined once excavation was completed and a comprehensive understanding of the stratification possible needs to be critically assessed. In some cases, the valid unit of archeological analysis remains the archeo-stratigraphic unit, in other cases it should be the geo-stratigraphic ensemble, and it may be necessary in all cases that the assemblages be trimmed into samples that exclude items collected in areas where inter-level displacement had a significant impact. This approach is not novel, and the organization of finds into “Archeological Horizons” whose composition is informed by both stratigraphic provenience and refitting-aided taphonomic reasoning has been used in the study of both Upper Paleolithic (e.g., Geissenklösterle [20] or Le Piage [21]) and Neolithic (e.g., Gruta do Caldeirão [63]) cave and rock-shelter sites.

Given the ubiquitous nature of the period’s reduction methods, applying this approach to the Middle Paleolithic would seem to be equally, if not more advantageous. Yet, with few exceptions [32,35–36], that is seldom the case, mostly due to the time-consuming nature of refitting analyses [64], or to the inadequacy of the data. The latter is especially the case when dealing with old collections, produced by outdated excavation methods, often on-site sorted (i.e., with discard of the finds devoid of perceived significance), and for which spatial and stratigraphic information with the necessary detail may be lacking—e.g., the Passemard excavations at Olha and Isturitz or the Peyrony excavations at Le Moustier and La Ferrassie [65–68].

The limited size of the excavation trenches may also prevent the application of a lithic taphonomy approach. The cave site of Gatzarria (Pyrénées-atlantiques, France), excavated by G. Laplace, is a case in point. Here, the Middle Paleolithic deposit is only known after two test trenches separated by several meters, which hinders the search for refitting links [69–71]. It can also be the case that, when at all re-excavated, old sites are the target of operations limited to the opening of small trenches or the rejuvenation of extant cross-sections, which poses a similar problem (see [72] for an exception). Yet, many recent excavations of Middle Paleolithic stratified sites are amenable to lithic taphonomy analysis, even though the definition of the analytical units used in the study of the stone tools often remains based solely on the geoarcheological unit of provenience.

Thus, our understanding of the Middle Paleolithic is currently affected by a methodological paradox. On one hand, most sites to which systematic stone tool refitting has been applied to assess issues of site formation and assemblage integrity are open-air ones. On the other hand, the criteria upon which the explanation of variation has been framed (e.g. [48]) are derived from the study of assemblages from cave and rock-shelter sites lacking such a lithic taphonomy critique. This situation is the more unsatisfactory because, in the Middle Paleolithic, the definition of industries and technocomplexes is largely based on patterns of association between traits (technological or typological) that, taken one at a time, are susceptible of being found across extensive chronological spans. Moreover, post-depositional disturbance is, to a greater or lesser degree, a universal feature of all stratified sites (the more so in cave and rock-shelter contexts). Taphonomic analysis of the integrity of the lithics provenanced to the different units of a given sequence therefore should be a prerequisite for the assessment of their validity as units of analysis for the archeological study of the human occupations in the context of which the stone tools were discarded. Coupled with a much-needed improvement in the resolution of chronometric dating methods, the application of stone tool refitting and associated instruments of lithic taphonomy should constitute the backbone upon which to assess, or rebuild, the chronostratigraphic schemes upon which the diachronic variability of the European Middle Paleolithic is currently understood.

In Iberia, the value of this approach has been demonstrated at such sites as the rock-shelters of El Salt, Quebrada and Romaní. At Gruta da Oliveira, our refitting study has shown that the impact of inter-level displacement was limited and, with the application of the appropriate filters, can be largely eliminated. The thusly filtered samples are sufficiently large to provide for reliable analyses of diachronic change, while the better-than-average and indeed quite good preservation of specific units provides for the use of the entire sample in both technological and spatial analyses.

For Gruta da Oliveira specifically, we could conclude the following:

- Layer 22, layer 21 and layer 15 are valid units of analysis for the study of short-term activity in the cave (namely, of intra-site functional variation in relation to associated hearth features and as revealed by the position of knapping spots or the distribution of use-worn items and cut-marked or burnt animal bone), while use of layer 20 for the same purpose requires prior elimination from the sample of items derived from areas in which refits show that wall effects caused significant contamination by material derived from overlying units.
- Within the area of the cave sampled by the excavation trench, the 27-S Chamber was the main activity area during the accumulation of layer 15.
- For the study of long-term change across the sequence, two internally coherent assemblages can be contrasted, upper (layers 15–19) and lower (layers 20–22)—it being acceptable, given the refit links and if need be for the convenience of a larger sample size, that layers 23–25 be added to the layers 20–22 assemblage.
- During the time interval of late MIS 5 to early MIS 4 represented by the stratigraphic units analyzed, raw-material economy and technology shifted from the Levallois reduction of predominantly flint to the centripetal, mainly non-Levallois reduction of predominantly quartzite and quartz, the latter accompanied by the production and discard of bifaces and flake-cleavers.

## Supporting information

S1 Table Distribution per archeo-stratigraphic unit of provenience (layer) of individual items in Gruta da Oliveira (layers 15–27) refit sets. The nature of the refit links, the layer of provenience (LoP) assignment, and its rationale, are given for each set. The Mousterian Cone proveniences (layers 26–27) are highlighted. See main text for definitions.
(XLSX)

S2 Table Distribution per analytical unit of provenience (ensemble) of individual items in Gruta da Oliveira (layers 15–27) refit sets. The nature of the refit links, the ensemble of provenience (EoP) assignment, and its rationale, are given for each set. The Mousterian Cone proveniences (layers 26–27) are highlighted. See main text for definitions.
(XLSX)
